# Antarctic microbial protein-rich extracts with cryoprotective potential for cell and viral preservation

**DOI:** 10.3389/fmicb.2026.1800179

**Published:** 2026-04-28

**Authors:** Joana Camila Lopes, Oscar Bruna-Romero, Rubens Tadeu Delgado Duarte

**Affiliations:** 1 Laboratory of Molecular Ecology and Extremophiles, Department of Microbiology, Immunology and Parasitology, Federal University of Santa Catarina, Florianópolis, Brazil; 2 Postgraduate Program in Biotechnology and Biosciences, Federal University of Santa Catarina, Florianópolis, Brazil; 3 Laboratory of Applied Immunology, Department of Microbiology, Immunology and Parasitology, Federal University of Santa Catarina, Florianópolis, Brazil

**Keywords:** Antarctica, cryoprotection, extreme microbiology, microbial protein-rich extracts, viral stability

## Abstract

Antarctic microorganisms are naturally adapted to extreme cold and freezing conditions, often through the production of cryoprotective biomacromolecules. In this study, 22 Antarctic bacterial and yeast isolates were initially characterized based on morphological traits and molecular identification. The isolates were subsequently screened for freezing resistance at −80 °C, and four representative strains (*Rhodotorula* sp. C01, *Pedobacter* sp. BGS4005, *Psychrobacter* sp. P61, and *Salinibacterium* sp. P45) were selected for protein extraction based on their survival rates. Intracellular and extracellular protein-rich extracts were then evaluated for cryoprotective activity in prokaryotic (*Escherichia coli*), eukaryotic (HEK293 cells), and viral (adenovirus) systems after 30–60 days of storage. Morphological and molecular characterization revealed 11 species displaying three main cellular morphotypes (cocci, coccobacilli, and bacilli), with variation in cell size. In *E. coli*, intracellular protein-rich extracts increased post-freezing viability, with the intracellular extract from *Salinibacterium* sp. P45, at 0.1 mg mL^–1^, promoting an increase of approximately 3%. Extracellular protein-rich extracts from *Pedobacter* sp. BGS4005 and *Psychrobacter* sp. P61 further enhanced *E. coli* survival. In HEK293 cells, intracellular protein-rich extracts from *Salinibacterium* sp. P45 at 1 mg mL^–1^ exhibited cryoprotective activity, maintaining cell viability above 20% following freezing. In addition, adenovirus suspensions treated with intracellular protein-rich extracts maintained viral titers over 60 days at 4 °C, with the extract from *Psychrobacter* sp. P61 at 1 mg mL^–1^ showing no significant loss in viral titers. Together, these results highlight Antarctic microorganisms as a promising source of natural cryoprotective compounds and reinforce their relevance in extreme microbiology and cryopreservation biotechnology.

## 1 Introduction

Cryopreservation is a fundamental process in biotechnology; however, its efficiency remains limited by freezing-induced cellular damage, challenges associated with the thawing process, and the toxicity of conventional cryoprotectants such as dimethyl sulfoxide (DMSO) and glycerol (Verheijen et al., 2019; Zhang et al., 2022; Bennett et al., 2024). Inadequate or uncontrolled thawing can exacerbate cellular stress, promote ice recrystallization, and compromise post-thaw viability, representing a critical yet often underestimated bottleneck in cryopreservation protocols. Although DMSO and glycerol are widely used due to its high efficiency, its cytotoxic effects and adverse cellular responses restrict its application, particularly in sensitive cell types (Pal et al., 2012; Ekpo et al., 2022; Świêciło et al., 2024; Madiraju et al., 2026). In this context, natural biomacromolecules with antifreeze activity have emerged as promising alternatives or complementary agents for the development of safer and more efficient cryopreservation strategies (Rizzo and Lo Giudice, 2020; Shaw et al., 2023; Lopes et al., 2024a; Jin et al., 2026).

Antifreeze proteins (AFP) are protein-based biomacromolecules capable of inhibiting ice formation and growth, thereby protecting biological systems from freezing-induced damage (Gilbert et al., 2004; Rahman et al., 2019; Davies, 2022). Two key properties underlie their cryoprotective function: ice recrystallization inhibition (IRI), which prevents the growth of ice crystals during freeze–thaw cycles (Knight and Duman, 1986; Gilbert et al., 2004; Rahman et al., 2019; Gruneberg et al., 2021; Sun et al., 2022), and thermal hysteresis (TH), which lowers the freezing point of aqueous solutions, preventing ice formation (Barrett, 2001; Guo et al., 2012; Adar et al., 2023). These properties have supported the application of AFP across a wide range of biotechnological fields, including the preservation of cells, tissues, organs, gametes, embryos, and frozen foods, as well as applications in agriculture and materials engineering (Fan et al., 2002; Gwak et al., 2015; Correia et al., 2021; Liu et al., 2021; Eskandari et al., 2024; Lopes et al., 2024a; Meng et al., 2024; Short et al., 2024).

Extreme cold environments, such as Antarctica, harbor microbial communities highly adapted to freezing and subzero conditions (Duarte et al., 2012; Liang et al., 2022). Bacteria and fungi from these ecosystems exhibit diverse cold-adaptation strategies, including the production of cryoprotective substances such as exopolysaccharides and antifreeze proteins (Joseph et al., 2019; Ramasamy et al., 2023; Gonçalves et al., 2024; Uko et al., 2024). While AFP are key contributors to freeze tolerance, increasing evidence suggests that additional protein-based factors may act synergistically to enhance cryoprotection (Davies and Sykes, 1997; Koushafar et al., 1997; Eskandari et al., 2020; Lopes et al., 2024a; Nam et al., 2024). This supports the evaluation of protein-rich extracts rather than purified compounds when assessing functional cryoprotective activity.

Currently, the commercial use of antifreeze proteins relies predominantly on marine-derived AFP types I, II, and III, primarily extracted from Antarctic fishes (Davies and Hew, 1990; DeVries, 2020; Bogan et al., 2024; Leal et al., 2024). However, ethical concerns, scalability limitations, and restricted structural diversity have driven growing interest in microbial sources, including bacteria, fungi, and algae from polar regions (Raymond and Remias, 2019; Batista et al., 2020; Singh et al., 2021; Lopes et al., 2024b). Although numerous AFP-producing microorganisms have been described in Antarctica and the Arctic, most studies remain focused on bioprospecting and molecular characterization, leaving significant gaps regarding their functional performance in applied cryopreservation systems (Gwak et al., 2015; Lee J. et al., 2015; Kim et al., 2017; Muñoz et al., 2017; Khan et al., 2020; Liu et al., 2021; Nam et al., 2024).

Few studies have investigated the effects of AFP from Antarctic microorganisms on the cryopreservation of prokaryotic and eukaryotic cells. Preliminary studies using fish AFP types I and III demonstrated improved post-thaw cell viability (Kawahara et al., 2009; Sreter et al., 2022). Similarly, the antifreeze protein LeIBP from the Arctic fungus *Glaciozyma* sp. AY30 showed protective activity in different mammalian cell lines as well as in algal cells, highlighting its potential application in cell cryopreservation (Lee et al., 2012; Lee J. et al., 2015; Lee J. R. et al., 2015; Koh et al., 2015). In addition, the recombinant protein GaAFP, derived from the Antarctic yeast *Glaciozyma martinii*, exhibited low cytotoxicity in human intestinal epithelial cell models commonly used in bioavailability studies (Majewska et al., 2025). More recently, the antifreeze protein MaIBP_RIV from the Arctic bacterium *Marinomonas arctica* demonstrated effective cryoprotective activity at ultralow concentrations, significantly reducing DMSO requirements while maintaining cell viability, underscoring its translational potential for safer cryopreservation strategies (Liao et al., 2025).

Despite these advances, the effects of antifreeze proteins and related biomacromolecules on viral stability remain unexplored. The preservation of virus-based vaccines and viral vectors during storage and transport represents a major challenge in global distribution, particularly under temperature fluctuations that can compromise efficacy (Rexroad et al., 2002; Maheshwari et al., 2004; Tuladhar et al., 2012). While research efforts are underway to develop liquid vaccines stable at ambient temperatures, the application of AFP and protein-based cryoprotectants for viral preservation has not yet been investigated (Lee et al., 2017; Berg et al., 2021; Hare et al., 2024; Schnetzinger et al., 2024).

Given these challenges, this study evaluated the cryoprotective potential of protein-rich intracellular and extracellular extracts obtained from Antarctic bacteria and fungi. The extracts were assessed for their ability to enhance the cryopreservation of prokaryotic (*Escherichia coli*) and eukaryotic (HEK293) cells frozen at −18 °C, as well as to preserve recombinant adenovirus stability during storage at 4 °C. This work provides a functional proof of concept for the application of protein-based biomacromolecules produced by Antarctic microorganisms, reinforcing their relevance within extreme microbiology and cryopreservation biotechnology.

## 2 Materials and methods

### 2.1 Antarctic isolates

A total of 22 Antarctic microbial strains obtained from previous studies conducted by our research group were used in this work (Lopes et al., 2024b). All isolates were obtained from soil, moss, and permafrost samples collected from different locations on the Antarctic Peninsula, including the Collins Glacier, the Baranowski Glacier, and areas near the Russian Bellingshausen Station, located on King George Island. The strains were previously identified by partial sequencing of the 16S rRNA gene (bacteria) or the internal transcribed spacer (ITS) region (yeasts), as described by Lopes et al. (2024b). Stock cultures were preserved in 10% (v/v) glycerol at −80 °C. Prior to the experiments, isolates were reactivated and checked for purity on R2A medium and incubated at 25 °C.

### 2.2 Molecular and morphological characterization

Phylogenetic analysis was performed using partial 16S rRNA gene sequences (V3–V4 regions). Sequences were aligned against the full-length 16S rRNA gene of *Escherichia coli* K-12 (LT899983) using MUSCLE v3.8.31, and non-aligned terminal regions were trimmed. Phylogenetic trees were constructed in ARB software (Ludwig et al., 2004) using the Maximum Likelihood method with Felsenstein correction and 1,000 bootstrap replicates. *Methanosarcina thermophila* DSM 1825 (AB973357) was used as the outgroup. Tree visualization and aesthetic adjustments were performed using FigTree v1.4.4.

Morphological characterization was carried out by scanning electron microscopy (SEM) at the Central Laboratory of Electron Microscopy (LCME), Federal University of Santa Catarina (UFSC). Isolates were cultivated on R2A medium until visible colony formation. Cells were harvested, fixed with 2.5% glutaraldehyde in 0.1 M sodium cacodylate buffer, post-fixed with 1% osmium tetroxide, dehydrated in a graded ethanol series (30%–100%), chemically dried with hexamethyldisilazane (HMDS), sputter-coated with gold (10 nm), and analyzed under an accelerating voltage of 15 kV. Cell length was measured using ImageJ software based on SEM scale bars, with three cells measured per isolate to obtain mean values.

### 2.3 Screening of freezing resistance at −80 °C

Freezing resistance at −80 °C was used exclusively as a screening criterion prior to cryopreservation assays conducted at −18 °C. All 22 Antarctic isolates were cultivated in R2B broth at 6 °C for 2 weeks in the dark without agitation. One milliliter of each culture was centrifuged at 10,000 × *g* for 3 min, and cell pellets were resuspended in 1 mL of phosphate-buffered saline (PBS).

Serial dilutions (up to 10^–6^) were prepared for initial colony-forming unit determination (N_0_). Cell suspensions were frozen at −80 °C for 24 h, thawed at room temperature for 30 min, and serially diluted again to determine post-thaw viable counts (N_1_). Aliquots were plated on R2A medium in triplicate. Survival rate (S) was calculated according to Kwon et al. (2018):


$$S\left(\%\right)=\frac{N_{1}}{N_{0}}\times 100$$

where “S” is the survival rate expressed as a percentage (%); “N_1_” is the viable cell density after thawing, and “N_0_” is the initial cell density (Kwon et al., 2018). Isolates exhibiting survival rates above 20% were considered freezing-resistant and selected for further analyses.

### 2.4 Selection of isolates for protein extraction

Isolates were classified into two groups based on freeze survival at −80 °C: high survival (>50%) and intermediate survival (20%–50%). From these groups, four representative strains were selected for protein extraction assays: *Pedobacter* sp. BGS4005 and *Salinibacterium* sp. P45 (high survival), and *Rhodotorula* sp. C01 and *Psychrobacter* sp. P61 (intermediate survival). Selection criteria included freezing tolerance, reproducible growth, biomass yield, and suitability for protein extraction.

### 2.5 Protein extraction from Antarctic bacteria and yeasts

Intracellular protein-rich extraction from bacterial isolates was performed as described by Gilbert et al. (2004). Briefly, bacterial strains were cultivated in R2B broth for 2 weeks at 15 °C and 4 °C with agitation (200 rpm) (Gilbert et al., 2004). Cultures were centrifuged at 5,000 × *g* for 15 min at 4 °C, and cell pellets were frozen at −18 °C to facilitate lysis. Thawed biomass (1 g) was treated with B-PER reagent (Thermo Fisher Scientific), lysozyme (50 mg mL^–1^), and DNase (≥ 2,500 U mL^–1^), incubated for 15 min at room temperature, and centrifuged at 5,000 × *g* for 15 min. Supernatants were collected as intracellular protein extracts and stored at −18 °C.

Yeast intracellular protein-rich extraction followed the protocol described by Mouro (2016). Yeasts were grown in R2B broth for 2 weeks at 15 °C and 4 °C with agitation. After centrifugation (5,000 × *g*, 15 min, 4 °C), biomass was frozen, thawed, standardized to 1 g, washed with buffer A (100 mM MOPS-NaOH, pH 6.8), and resuspended in buffer B (100 mM MOPS, pH 6.8, containing 20% glycerol, 0.5 mM EDTA, and 0.5 M DTT). Glass beads were added, and samples were vortexed for 5 min, followed by centrifugation at 12,000 × *g* for 5 min. Supernatants were collected as intracellular extracts and stored at −18 °C.

Protein concentrations were quantified using the Bradford assay for standardization in cryopreservation assays. Extracellular protein-rich extracts were obtained by collecting culture supernatants following centrifugation and were used without concentration standardization to preserve their native composition. All extraction procedures were performed under sterile conditions in a laminar flow biosafety cabinet using sterile materials to minimize contamination.

### 2.6 Cryoprotective activity in prokaryotic cells

The cryoprotective assays were performed at −18 °C because freezing at this temperature typically results in slower freezing rates and the formation of larger ice crystals, which can cause greater cellular damage. Therefore, this condition represents a more stringent model to evaluate the protective effects of microbial protein-rich extracts against freezing stress.

The cryoprotective activity of microbial protein-rich extracts was evaluated using *E. coli* ATCC 25922. Cells were grown in TSB medium at 25 °C with agitation (200 rpm) until reaching an OD_600_ of 0.8–1.0. Aliquots were centrifuged at 10,000 × *g* for 3 min, washed twice with PBS, and resuspended in intracellular protein-rich extracts at concentrations of 1 and 0.1 mg mL^–1^, or in extracellular extracts.

Cell suspensions were frozen at −18 °C for 24 h, thawed, serially diluted, and plated for CFU determination. Controls included untreated cells, 1 mg mL^–1^ type III antifreeze protein, and protein-inactivated treatments using proteinase K (2.75 mg mL^–1^, 25 °C, 30 min) or heat treatment (100 °C, 30 min).

### 2.7 Cryoprotective activity in eukaryotic cells

HEK293 cells were cultured in DMEM supplemented with 10% (v/v) fetal bovine serum at 37 °C and 5% CO_2_. At approximately 90% confluency, cells were harvested, counted using Trypan Blue exclusion, and frozen at −18 °C for 30 days in the presence of the following cryoprotectants: 1 mg mL^–1^ microbial protein-rich extracts, 10% DMSO (reference control), 1 mg mL^–1^ type III AFP (positive control), or 40% fetal bovine serum (negative control). Samples were placed directly into a −18 °C freezer without the use of a controlled-rate freezing container or freezing support system. Post-thaw viability was determined using Trypan Blue staining (see the Supplementary Material 1).

### 2.8 Evaluation of adenovirus stability at 4 °C

Recombinant non-replicative adenovirus (RAdCMV-CE1) was produced in HEK293 cells as described by Bruña-Romero et al. (1997). Viral stocks were extracted, purified using Vivapure^®^ Adenopack™ 500 RT columns, and stored at −18 °C. Stabilizing formulations containing microbial protein-rich extracts were mixed with viral suspensions (1:1, v/v) and stored at 4 °C for up to 60 days (see the Supplementary Material 2). Viral stability was evaluated by titration at 0, 15, 30, 45, and 60 days using limiting dilution assays in HEK293 cells. Viral titers were expressed as plaque-forming units (PFU).

### 2.9 Statistical analysis

Data were assessed for normality using the Kolmogorov–Smirnov test and for homogeneity of variances using the Cochran test. Differences among treatments were analyzed by one-way analysis of variance (ANOVA), followed by the Student–Newman–Keuls (SNK) *post-hoc* test when appropriate. Statistical significance was set at *p* < 0.05. Statistical analyses were performed to compare freezing resistance, bacterial survival, eukaryotic cell viability, and viral stability across treatments.

## 3 Results

### 3.1 Molecular and morphological characterization of Antarctic isolates

**FIGURE 1:**
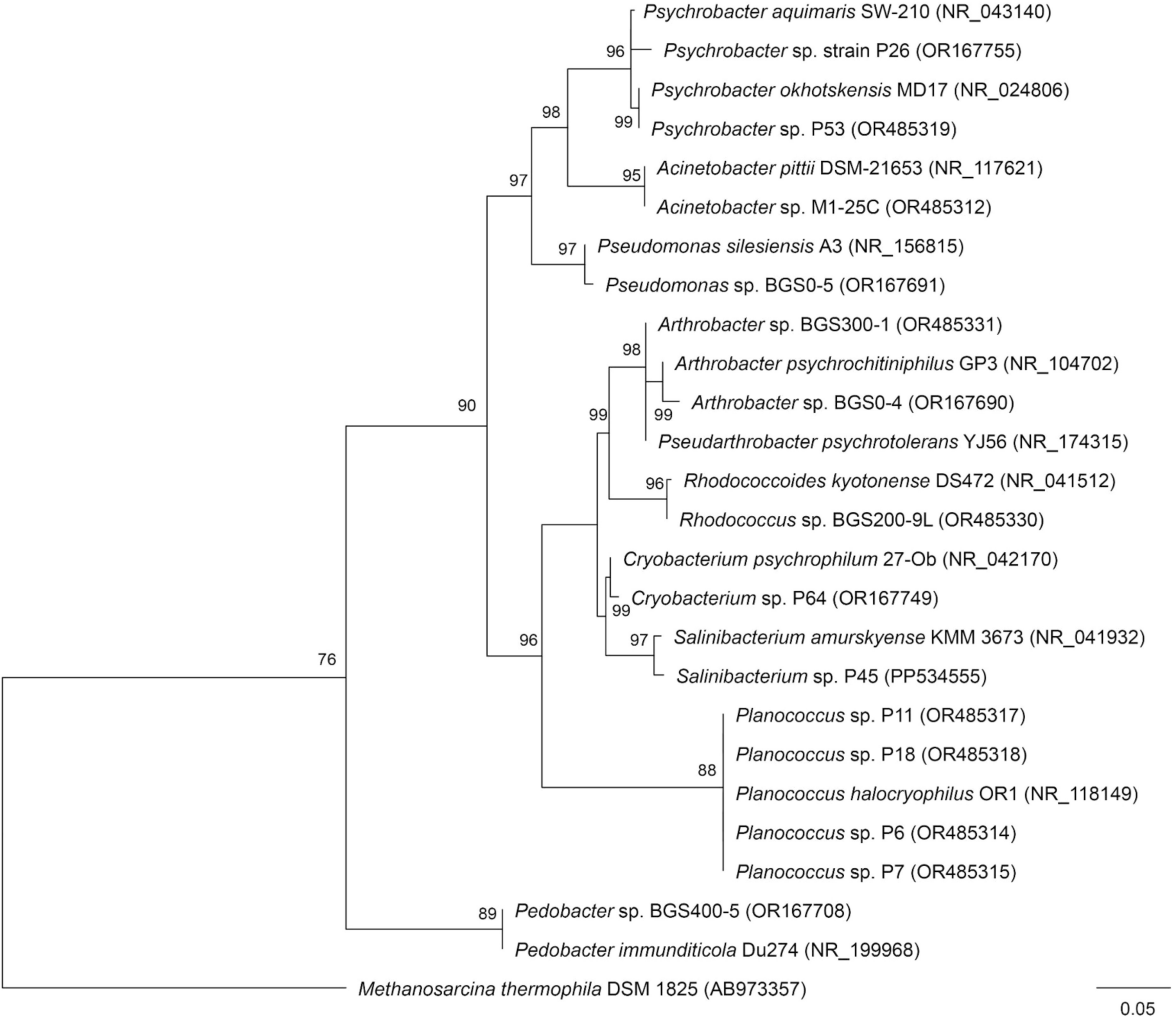
Phylogenetic tree of Antarctic isolates based on the 16S rRNA gene sequence.

Fourteen representative sequences derived from the 22 Antarctic isolates were selected for phylogenetic analysis based on sequence quality and taxonomic diversity. These sequences represented 11 distinct taxa and were used to construct the phylogenetic tree (Figure 1). Comparative analysis of partial 16S rRNA gene sequences against the GenBank database indicated that all isolates were affiliated with known bacterial genera, showing sequence similarities ranging from 85% to 99%.

The strains *Psychrobacter* sp. P26 and *Psychrobacter* sp. P61 showed highest similarity to *Psychrobacter aquimaris* (96%) and Psychrobacter okhotskensis (99%), respectively. *Acinetobacter* sp. M125C was most closely related to *Acinetobacter pittii* (95%), while *Pseudomonas* sp. BGS05 showed 97% similarity to *Pseudomonas silesiensis*. The strains *Arthrobacter* sp. BGS04 and BGS3001 were affiliated with *Arthrobacter psychrochitiniphilus* (99%) and *Pseudarthrobacter psychrotolerans* (98%), respectively. *Rhodococcus* sp. BGS2009L showed 96% similarity to *Rhodococcoides kyotonense*, and *Cryobacterium* sp. P64 showed 99% similarity to *Cryobacterium psychrophilum*. *Salinibacterium* sp. P45 was affiliated with *Salinibacterium amurskyense* (97%). Strains of the genus *Planococcus* (P18, P11, P6, and P7) showed closest similarity to *Planococcus halocryophilus* (88%). *Pedobacter* sp. BGS4005 showed highest similarity to *Pedobacter immunditicola* (89%).

Scanning electron microscopy revealed marked morphological diversity among the Antarctic isolates (Table 1; see Supplementary Material 3). Bacterial cells exhibited coccoid, cocco-bacillary, and rod-shaped morphologies. Cocci accounted for approximately 55% of the observed forms, rods for 38%, and cocco-bacilli for 5%. Members of the genera *Planococcus* and *Arthrobacter* exhibited coccoid morphology, *Psychrobacter* displayed both cocco-bacillary and rod-shaped forms, *Acinetobacter* appeared predominantly cocco-bacillary, while *Salinibacterium*, *Pedobacter*, *Cryobacterium*, *Rhodococcus*, *Phyllobacterium*, and *Pseudomonas* were rod-shaped. Yeast isolates of the genus *Rhodotorula* exhibited elongated cells ranging from 2.317 to 3.056 μm in length. Bacterial cell lengths varied substantially, with *Planococcus* isolates representing the smallest cells (0.249–0.760 μm) and *Pseudomonas* isolates reaching lengths of up to 1.304 μm, highlighting the morphological diversity of the Antarctic microbial community.

**TABLE 1:** Antarctic microbial isolates used in this study, including isolate code, taxonomic identification based on 16S rRNA gene sequencing for bacteria and internal transcribed spacer (ITS) region sequencing for fungi, GenBank accession numbers, percentage of sequence identity, source of isolation, and average cell length (μm) determined by electron microscopy.

Code	Identification	GenBank number	Id percentage (%)	Source	Length (μm)
C01	*Rhodotorula* sp.	OR237961	100	Collins Glacier soil - west face	3.056
C1001	*Rhodotorula* sp.	OR339876	99.80	Collins Glacier soil - west face	2.317
P6	*Planococcus* sp.	OR485314	100	Permafrost	0.660
P9	*Planococcus* sp.	OR485316	100	Permafrost	0.760
P11	*Planococcus* sp.	OR485317	100	Permafrost	0.418
P18	*Planococcus* sp.	OR485318	100	Permafrost	0.268
P25	*Planococcus* sp.	OR167740	96.85	Permafrost	0.249
P7	*Planococcus* sp.	OR485315	100	Permafrost	0.600
P26	*Psychrobacter* sp.	OR167755	99.44	Marine sediment	0.989
P45	*Salinibacterium* sp.	PP534555	98.74	Marine sediment	1.028
P49	*Salinibacterium* sp.	OR167753	100	Marine sediment	0.922
P53	*Psychrobacter* sp.	OR485319	100	Permafrost	0.995
P61	*Psychrobacter* sp.	OR167757	99.65	Marine sediment	0.417
P64	*Cryobacterium* sp.	OR167749	98.32	Marine sediment	0.389
M1_25C	*Acinetobacter* sp.	OR485312	99.6	Collins Glacier moss - west face	0.689
BGS04	*Arthrobacter* sp.	OR167690	99.46	Baranowski Glacier soil	0.598
BGS05	*Pseudomonas* sp.	OR167691	99.72	Baranowski Glacier soil	1.304
BGS2009L	*Rhodococcus* sp.	OR485330	99.52	Baranowski Glacier soil	1.268
BGS3001	*Arthrobacter* sp.	OR485331	99.85	Baranowski Glacier soil	0.575
BGS4005	*Pedobacter* sp.	OR167708	98.74	Baranowski Glacier soil	0.780
PSC253	*Psychrobacter* sp.	OR485320	100	Collins Glacier soil – southeast face	0.680
P31	*Phyllobacterium* sp.	OR167751	96.88	Marine sediment	1.024

### 3.2 Screening of freezing resistance at −80 °C

**FIGURE 2:**
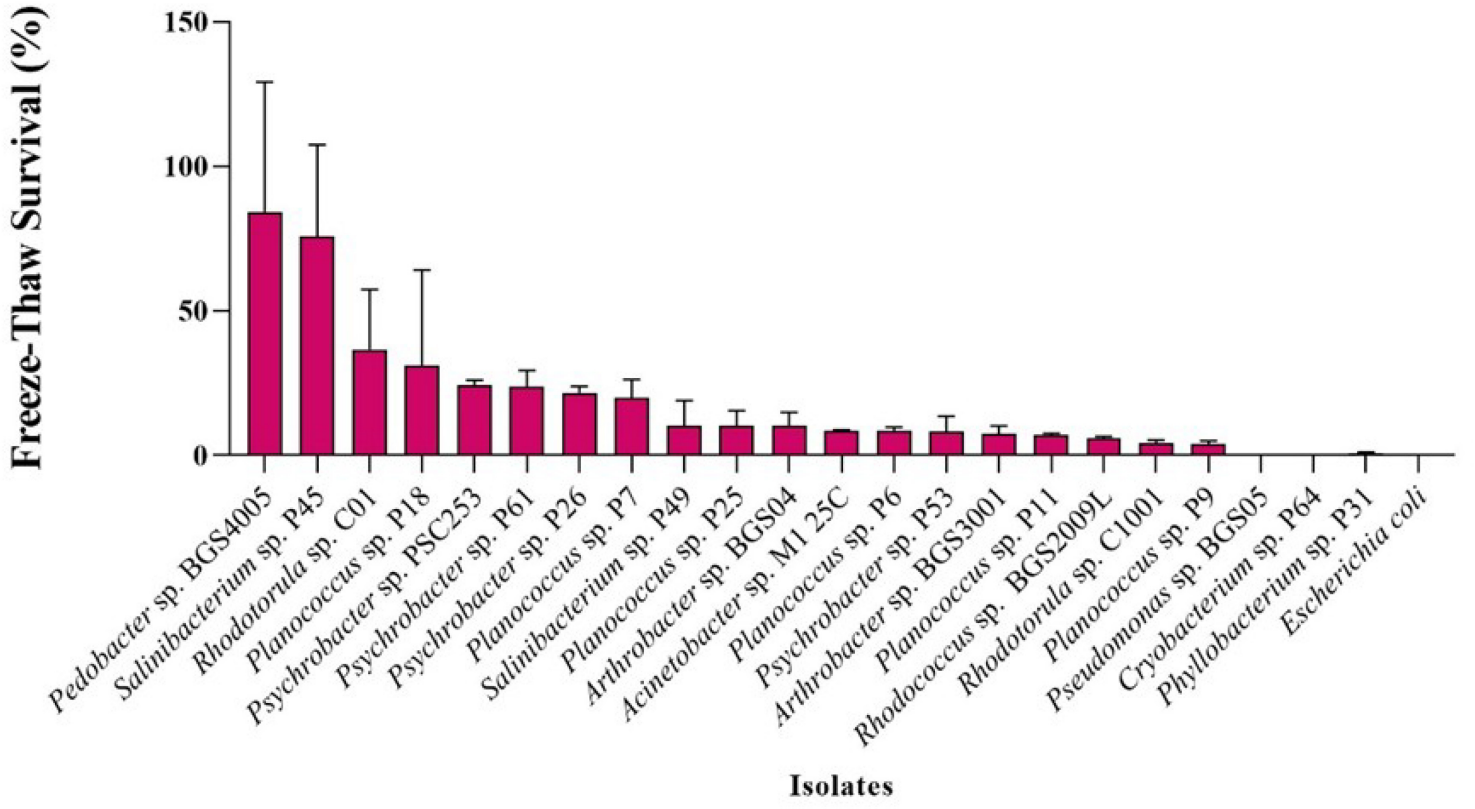
Freeze-thaw (FT) survival rate at –80 °C of Antarctic isolates. The experiments were conducted in replicate.

All 22 Antarctic isolates were evaluated for freezing resistance following exposure to −80 °C (Figure 2). Survival rates varied widely among the isolates. High survival rates (>50%) were observed for *Pedobacter* sp. BGS4005 (84.12%) and *Salinibacterium* sp. P45 (75.80%). Intermediate survival rates (20%–50%) were observed for *Rhodotorula* sp. C01 (36.66%) and *Planococcus* sp. P18 (31.18%), *Psychrobacter* sp. PSC253 (24.44%), *Psychrobacter* sp. P61 (23.88%), *Psychrobacter* sp. P26 (21.62%), and *Planococcus* sp. P7 (20.02%).

Several isolates showed low survival rates (<10%), including *Salinibacterium* sp. P49, *Planococcus* sp. P25, *Arthrobacter* sp. BGS04, *Acinetobacter* sp. M125C, *Planococcus* sp. P6, *Psychrobacter* sp. P53, *Arthrobacter* sp. BGS3001, *Planococcus* sp. P11, *Rhodococcus* sp. BGS2009L, *Rhodotorula* sp. C1001, and *Planococcus* sp. P9. No viable cells were detected after freezing for *Pseudomonas* sp. BGS05, *Cryobacterium* sp. P64, or *Phyllobacterium* sp. P31.

### 3.3 Cryoprotective activity of microbial proteins in prokaryotic cells

**FIGURE 3:**
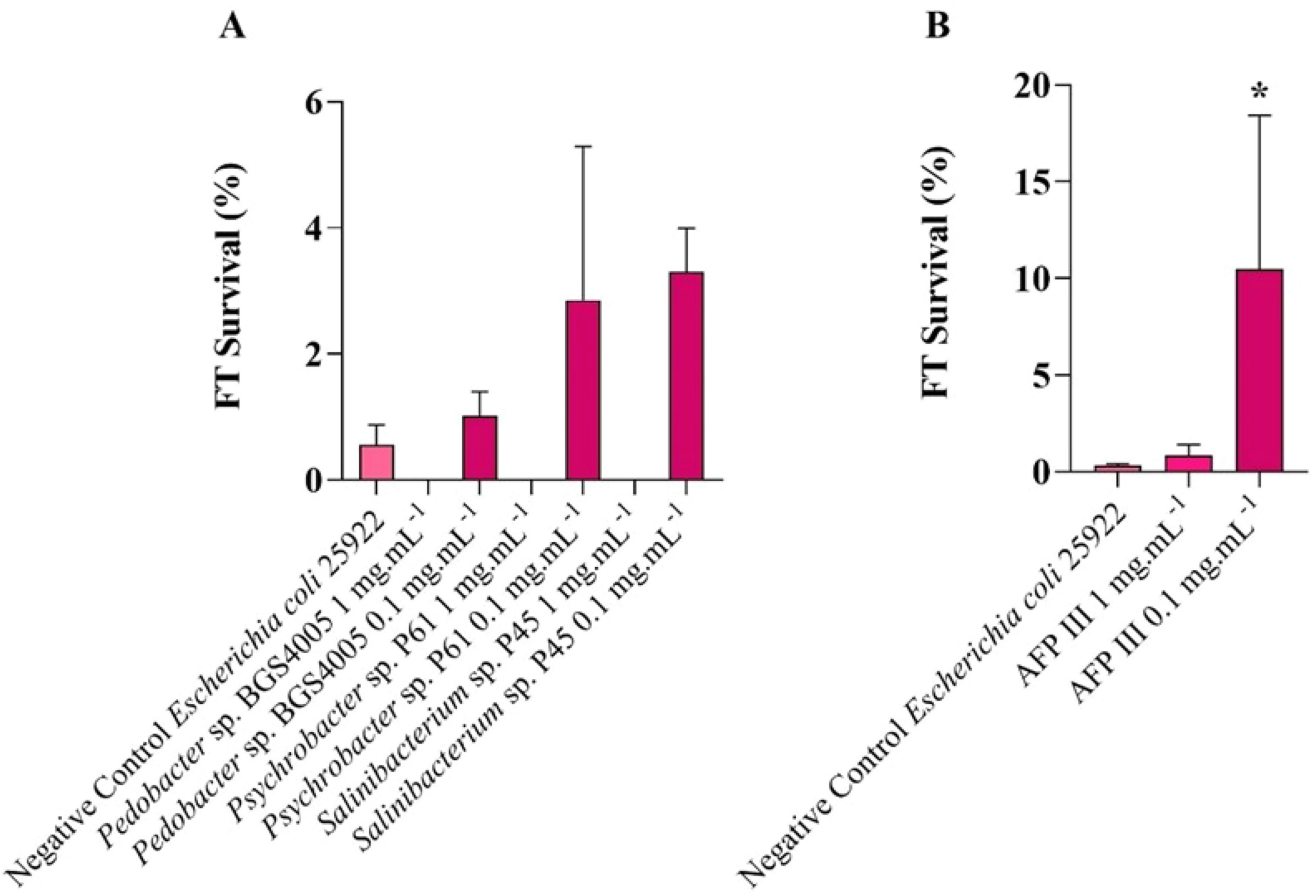
Relationship between the survival rate freeze-thaw (FT) of *Escherichia coli* after freezing at –18 °C under different treatments of total intracellular protein-rich extracts from Antarctic isolates **(A)** and under different concentrations of antifreeze proteins (AFP) type III **(B)**. The experiments were conducted in triplicate. An asterisk (*) indicates significant diferences between the treatment and negative control.

The cryoprotective activity of intracellular protein-rich extracts obtained from *Pedobacter* sp. BGS4005, *Psychrobacter* sp. P61, and *Salinibacterium* sp. P45 was evaluated using *E. coli* as a model prokaryotic system. At a concentration of 1 mg mL^–1^, none of the intracellular protein-rich extracts significantly improved *E. coli* survival after freezing at −18 °C, showing survival rates comparable to the untreated control (*p* > 0.05) (Figure 3A).

In contrast, intracellular protein-rich extracts tested at 0.1 mg mL^–1^ significantly enhanced *E. coli* survival compared to the negative control (*p* < 0.05) (Figure 3B). Survival rates reached 1.0% for *Pedobacter* sp. BGS4005, 2.85% for *Psychrobacter* sp. P61, and 3.3% for *Salinibacterium* sp. P45. Type III antifreeze protein (AFP III) showed no cryoprotective effect at 1 mg mL^–1^, whereas significant protection was observed at 0.1 mg mL^–1^, indicating a concentration-dependent effect.

**FIGURE 4:**
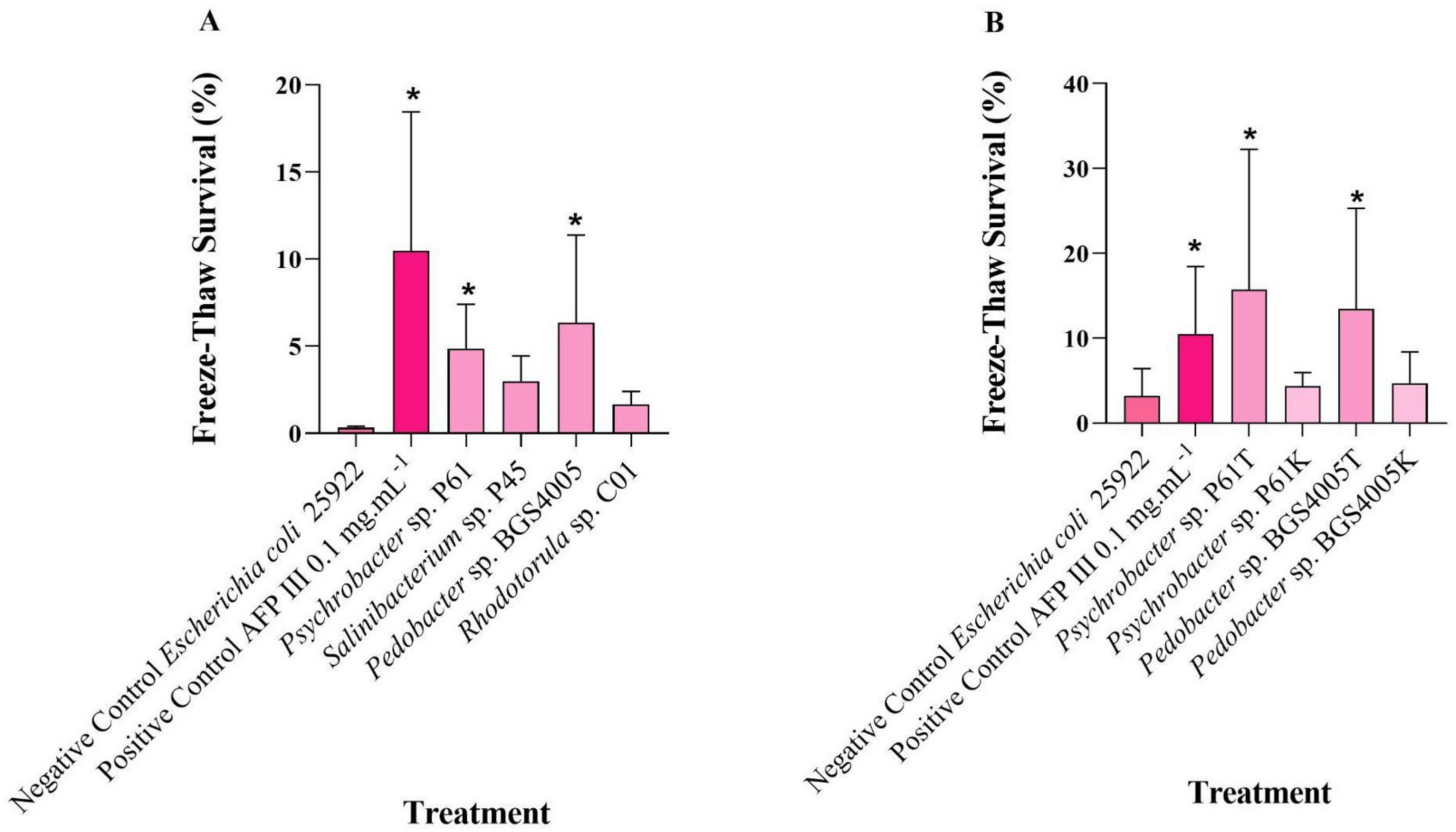
Survival rate freeze-thaw (FT) of *Escherichia coli* to freezing at −18 °C under different treatments with: extracellular protein-rich extracts from Antarctic isolates **(A)** and extracellular protein-rich extracts from Antarctic isolates subjected to thermal and enzymatic treatment with proteinase K 2.75 mg mL^–1^ **(B)**. The experiments were conducted in triplicate. On both figures, an asterisk (*) indicates significant differences between the treatment and negative control.

Extracellular protein-rich extracts from *Pedobacter* sp. BGS4005, *Psychrobacter* sp. P61, *Salinibacterium* sp. P45, and *Rhodotorula* sp. C01 were also evaluated (Figure 4A). The untreated control exhibited a survival rate of 0.33%, while AFP III (1 mg mL^–1^) resulted in a survival rate of 0.84%. Among microbial protein-rich extracts, *Pedobacter* sp. BGS4005 showed the highest cryoprotective effect (5.51%), followed by *Psychrobacter* sp. P61 (4.61%), *Salinibacterium* sp. P45 (2.51%), and *Rhodotorula* sp. C01 (1.64%).

To confirm the proteinaceous nature of the cryoprotective effect, extracellular protein-rich extracts from *Pedobacter* sp. BGS4005 and *Psychrobacter* sp. P61 were subjected to heat treatment (100 °C for 30 min) or enzymatic digestion with proteinase K (2.75 mg mL^–1^, 30 min). Heat treatment did not significantly affect the cryoprotective activity of the extracts. In contrast, proteinase K digestion significantly reduced the cryoprotective effect compared to untreated extracts (*p* < 0.05) (Figure 4B). These results indicate that the cryoprotective activity is mediated by extracellular proteins, which are resistant to high temperatures.

### 3.4 Cryoprotective activity of microbial proteins in eukaryotic cells

**FIGURE 5:**
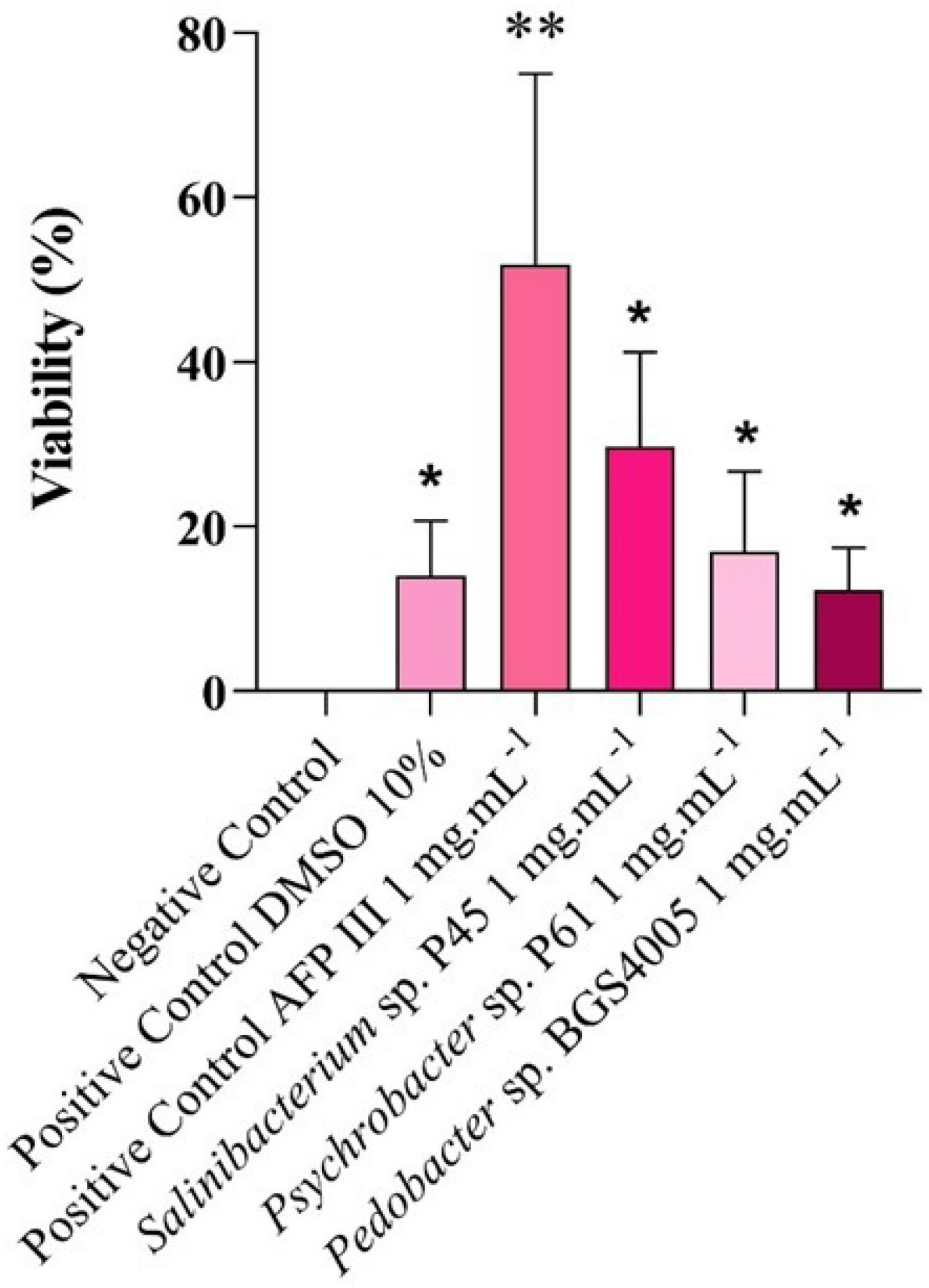
Analysis of cell viability using trypan blue in HEK293 cells after freezing for 30 days at –18 °C. A total of 5 × 10^6^ cells was cryopreserved with 10% dimethyl sulfoxide (DMSO), 1 mg mL^– 1^ of fish antifreeze protein type III, and protein-rich extracts obtained from Antarctic bacteria. The experiment was conducted in triplicate. An asterisk (*) indicates significant differences compared to the negative control. A double asterisk (**) indicates significant diferences between the treatment and other treatments.

The cryoprotective effects of microbial protein-rich extracts on HEK293 cells are shown in Figure 5. No viable cells were detected in the untreated control. Cells frozen in the presence of 10% DMSO (reference cryoprotectant) exhibited a post-thaw viability of approximately 15%. Treatment with type III AFP (1 mg mL^–1^) resulted in significantly higher cell viability (51.85%) compared to all other treatments (*p* < 0.05).

Intracellular protein-rich extracts from *Salinibacterium* sp. P45, *Psychrobacter* sp. P61, and *Pedobacter* sp. BGS4005, tested at 1 mg mL^–1^, resulted in cell viabilities of approximately 29.75%, 17%, and 12.25%, respectively. No significant differences were observed between these treatments and the DMSO reference (*p* > 0.05), indicating that microbial protein-rich extracts provided cryoprotection comparable to that of DMSO in HEK293 cells.

### 3.5 Cryoprotective activity of microbial proteins in adenovirus stability

**FIGURE 6:**
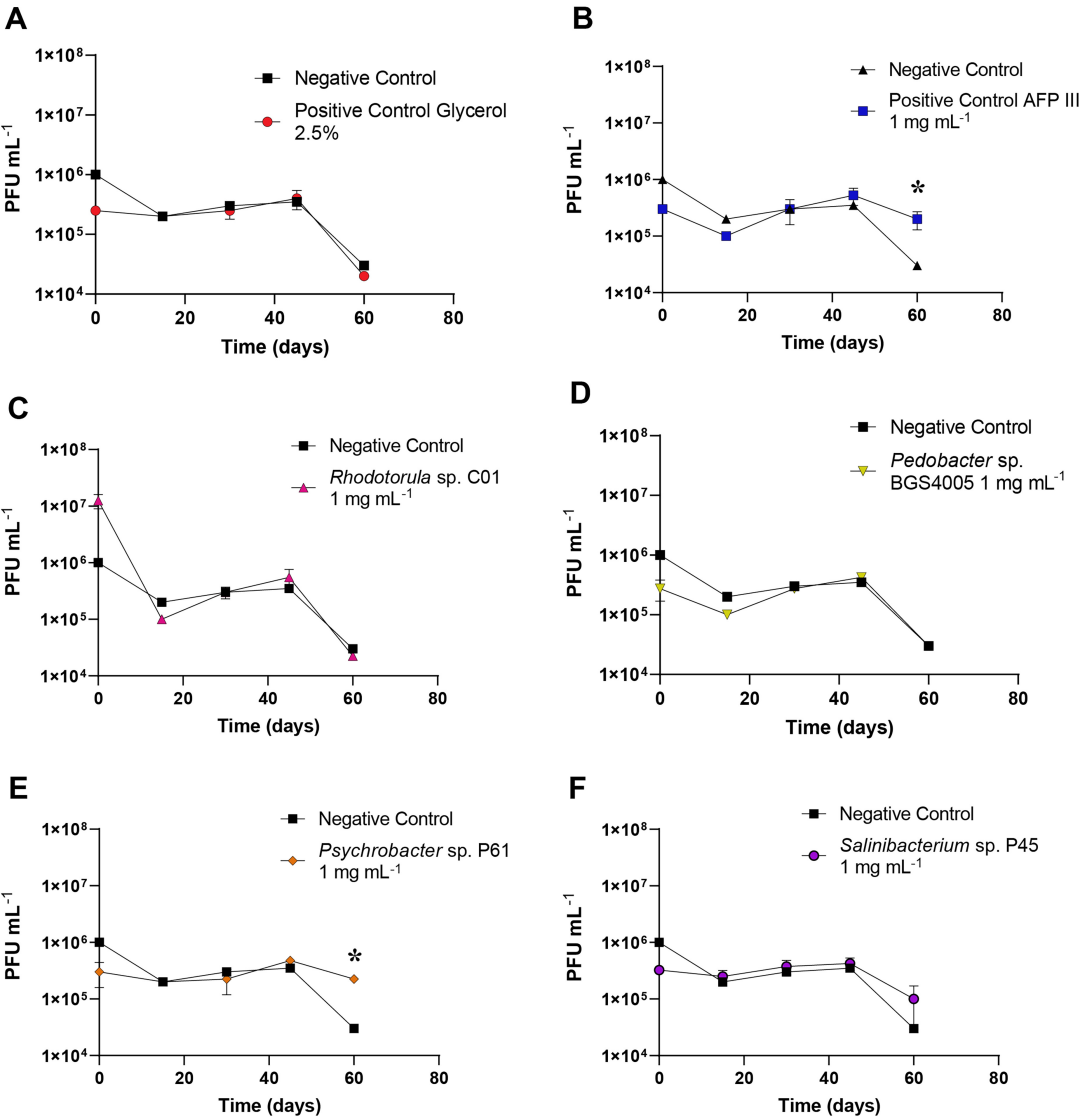
Evaluation of adenovirus stability over 60 days treated with: Glycerol 2.5% **(A)**, AFP type III 1 mg mL^– 1^ **(B)**, *Rhodotorula* sp. C01 1 mg mL^– 1^ **(C)**, *Pedobacter* sp. BGS4005 1 mg mL^– 1^ **(D)**, *Psychrobacter* sp. P61 1 mg mL^– 1^ **(E)**, and *Salinibacterium* sp. P45 1 mg mL^– 1^ **(F)**. The experiment was conducted in duplicate. An asterisk (*) indicates significant differences for the PFU concentration between the treatment and the negative control at the respective time.

The stability of recombinant adenovirus during storage at 4 °C was evaluated over a 60-day period in the presence of microbial protein-rich extracts (Figure 6). Viral titers remained stable across all treatments during the first 45 days, with no significant differences observed relative to the negative control (*p* > 0.05).

After 60 days, a significant reduction in viral titers was observed in the negative control, the glycerol-treated control, and treatments containing protein-rich extracts from *Rhodotorula* sp. C01, *Pedobacter* sp. BGS4005, and *Salinibacterium* sp. P45. In contrast, viral suspensions are treated with the total protein-rich extract from *Psychrobacter* sp. P61 showed no significant loss in viral titers over the storage period and differed significantly from the negative control (*p* < 0.05). Type III AFP also preserved viral titers and showed a significant protective effect compared to the negative control (*p* < 0.05).

These results demonstrate that protein-based biomacromolecules produced by Antarctic microorganisms, particularly *Psychrobacter* sp. P61, effectively enhances long-term adenovirus stability, highlighting their potential application in viral preservation strategies.

## 4 Discussion

Antarctic microorganisms represent an important yet still underexplored source of cryoprotective biomacromolecules with significant biotechnological potential (Liang et al., 2022; Shaw et al., 2023). In polar environments, microorganisms are exposed to extreme selective pressures, including persistent subzero temperatures, repeated freeze–thaw cycles, limited nutrient availability, and prolonged periods of water unavailability (Holmberg and Jørgensen, 2023). To survive under such conditions, these organisms have evolved a diverse set of adaptive strategies, including the production of antifreeze proteins (AFPs), exopolysaccharides, and other cold-adapted biomolecules capable of mitigating freezing-induced cellular damage (Ramasamy et al., 2023).

Phylogenetic analysis based on 16S rRNA gene sequences revealed that the Antarctic isolates investigated in this study belong predominantly to genera commonly associated with cold environments, such as *Psychrobacter*, *Cryobacterium*, *Planococcus*, *Arthrobacter*, and *Pedobacter* (Alam et al., 2003; Kanekar and Kanekar, 2022; Masnoddin et al., 2022; Decewicz et al., 2023; Teoh et al., 2024; Wood et al., 2025). Many of these genera are well-known for their psychrophilic or psychrotolerant characteristics and have been repeatedly reported in polar and alpine ecosystems. The high sequence similarity observed for most isolates reinforces their adaptation to low-temperature environments. Conversely, several isolates exhibited similarity values below the conventional 97% threshold, particularly within *Planococcus* and *Pedobacter*, suggesting the presence of genetically distinct lineages that may be underrepresented in public databases. These findings are consistent with previous reports describing polar regions as reservoirs of unique microbial diversity shaped by strong environmental selection pressures (de Pascale et al., 2012; Zucconi et al., 2025).

The morphological diversity observed among the isolates further reflects their adaptive strategies. The predominance of coccoid and small rod-shaped bacteria may confer increased resistance to physical stresses such as freezing and desiccation, while variability in cell size suggests morphological plasticity as an additional mechanism for coping with environmental constraints. The small cell dimensions observed in *Planococcus* isolates may enhance metabolic efficiency in oligotrophic and cold habitats by increasing the surface-area-to-volume ratio (Mykytczuk et al., 2013). In contrast, yeasts of the genus *Rhodotorula* exhibited larger cell sizes, consistent with previous studies, and potentially linked to enhanced cellular robustness and maintenance of metabolic activity under thermal stress (Kan et al., 2025). Collectively, these observations indicate a close relationship between cellular morphology and environmental adaptation in Antarctic microorganisms.

Assessment of freeze resistance at −80 °C revealed substantial variability among the 22 tested isolates, highlighting that freezing tolerance is not uniformly distributed across taxa or even within the same genus. High survival rates were observed for *Pedobacter* sp. BGS4005, *Salinibacterium* sp. P45, *Rhodotorula* sp. C01, and *Planococcus* sp. P18, whereas several isolates showed minimal or no survival. Intermediate resistance was observed in multiple *Psychrobacter* strains, including P61 and P26, while others, such as *Psychrobacter* sp. P53, exhibited low tolerance. This intra-genus variability likely reflects differences in genomic composition, metabolic flexibility, and the capacity to synthesize cryoprotective compounds or modify membrane structure in response to freezing stress (Walker et al., 2006; Wilson et al., 2006, 2012).

These results align with previous studies reporting freezing resistance in Antarctic microorganisms, including members of *Arthrobacter*, *Psychrobacter*, and *Rhodococcus*, and support the notion that certain genera possess more efficient protective mechanisms against extreme freezing (Moreira et al., 2022). Importantly, resistance to freezing at −80 °C should be interpreted primarily as a selection criterion, identifying microorganisms capable of surviving extreme cold, rather than as a direct indicator of cryoprotective efficacy in applied systems.

Indeed, subsequent experiments demonstrated that freeze resistance does not necessarily correlate with cryoprotective performance in heterologous systems. Intracellular protein-rich extracts tested for the cryopreservation of *E. coli* at −18 °C exhibited a clear concentration-dependent effect. At 1 mg mL^–1^, intracellular protein-rich extracts were ineffective or even detrimental to cell survival, whereas lower concentrations (0.1 mg mL^–1^) significantly increased post-thaw viability. This non-linear relationship is consistent with previous observations for purified AFPs, where higher concentrations have been associated with bactericidal or inhibitory effects, possibly due to membrane disruption or interference with essential cellular processes (Kawahara et al., 2009). These findings emphasize that optimal cryoprotection depends not only on the presence of antifreeze activity but also on appropriate dosage.

Extracellular protein-rich extracts displayed a more pronounced cryoprotective effect on *E. coli* cells. Among the tested isolates, the extracellular protein-rich extract from *Pedobacter* sp. BGS4005 yielded the highest survival rates, outperforming both the negative control and the AFP type III positive control. Extracts protein-rich from *Psychrobacter* sp. P61, *Salinibacterium* sp. P45, and *Rhodotorula* sp. C01 also enhanced bacterial survival, suggesting that secreted biomolecules contribute significantly to cryoprotection. Enzymatic degradation with proteinase K markedly reduced the cryoprotective activity, whereas thermal treatment did not significantly affect this effect, indicating that extracellular proteins play a central role in mediating the observed cryoprotection.

The ability of *Pedobacter*, *Psychrobacter*, and *Rhodotorula* species to secrete cryoprotective biomolecules is well-supported by the literature, with several studies reporting the production of AFPs and exopolysaccharides in these genera (Gilbert et al., 2004; Singh et al., 2014; Yu et al., 2016; Hamidi et al., 2020; Moreira et al., 2022; Wang et al., 2023; Lopes et al., 2024b). Such extracellular compounds may act by inhibiting ice recrystallization, stabilizing cellular membranes, or modulating ice–water interfaces during freezing. However, in our previous study (Lopes et al., 2024b), the isolates *Pedobacter* sp. BGS4005, *Salinibacterium* sp. P45, *Psychrobacter* sp. P61 and *Rhodotorula* sp. C01 did not exhibit detectable ice recrystallization inhibition (IRI) activity when compared to the negative control. This suggests that the cryoprotective effects observed here may involve alternative mechanisms. For example, some antifreeze proteins are known to exhibit thermal hysteresis activity, which can influence ice crystal growth and freezing dynamics. Additionally, because the present study employed non-purified protein-rich extracts, it is possible that other biomolecules present in the mixture, such as polysaccharides or other extracellular components, contribute to cryoprotection through mechanisms including membrane stabilization or osmotic pressure regulation during freezing. However, these factors were not evaluated in the present study.

Cryoprotective efficacy was also evaluated in eukaryotic systems using HEK293 cells. Fish AFP type III significantly enhanced post-thaw viability, confirming its well-established role as a potent cryoprotectant (Sreter et al., 2022). Notably, bacterial protein-rich extracts exhibited cryoprotective effects comparable to 10% DMSO, suggesting their potential as less toxic alternatives for cell preservation (Majewska et al., 2025). These findings are consistent with previous reports demonstrating that AFP supplementation can reduce DMSO requirements while maintaining high cell viability across different mammalian cell types (Lee J. et al., 2015; Tomás et al., 2019; Liao et al., 2025).

It is important to note that the overall post-thaw viability observed in this study was relatively low, including for the 10% DMSO control (15%). This outcome likely reflects the experimental freezing conditions, in which cells were placed directly at −18 °C without controlled-rate freezing. Such conditions are known to promote slower freezing rates and the formation of larger ice crystals, which can cause increased cellular damage during freezing and thawing. Therefore, the low baseline viability should be interpreted in the context of this intentionally stringent freezing model. Furthermore, the use of protein-rich extracts may limit maximal efficacy, as the presence of non-functional or inhibitory components could interfere with optimal cryoprotection.

A particularly novel aspect of this study is the evaluation of Antarctic microbial proteins in viral preservation. Over a 60-day storage period, viral titers declined significantly in the negative control, glycerol-treated samples, and formulations containing protein-rich extracts from *Rhodotorula* sp. C01, *Pedobacter* sp. BGS4005, and *Salinibacterium* sp. P45. In contrast, viral suspensions treated with protein-rich extracts from *Psychrobacter* sp. P61 maintained stable viral titers and differed significantly from the negative control, demonstrating a protective effect comparable to fish AFP type III. Importantly, this stabilization reflects preservation of viral titers rather than infectivity enhancement, highlighting the role of these proteins in maintaining viral structural integrity during storage. Since the experiments were conducted at 4 °C, the mechanism involved is unlikely to be related to ice recrystallization inhibition. Instead, the stabilizing effect may be associated with the ability of these proteins to interact with viral particles, potentially preventing capsid aggregation or structural denaturation during storage. Given the growing interest in thermostable vaccine formulations, these findings underscore the potential application of Antarctic microbial proteins in viral and vaccine preservation strategies (Berg et al., 2021; Mostafa et al., 2024).

Despite these promising results, the use of protein-rich extracts represents a key limitation of the present study. These extracts contain complex mixtures of proteins and other biomolecules, some of which may counteract cryoprotective activity. Future work should focus on purification and molecular characterization of the active components, enabling precise dose optimization and mechanistic insights. Additionally, combining microbial AFPs with other cryoprotectants, such as reduced concentrations of DMSO, may further enhance cryopreservation outcomes (Sreter et al., 2022; Nam et al., 2024; Liao et al., 2025).

In conclusion, this study demonstrates that Antarctic microorganisms constitute a valuable source of cryoprotective biomacromolecules with applications spanning microbial preservation, mammalian cell cryopreservation, and viral stability. High freeze resistance observed in selected strains, combined with the demonstrated efficacy of microbial protein-rich extracts, highlights new opportunities for developing less toxic and more effective cryoprotective formulations. These findings reinforce the biotechnological relevance of polar microorganisms and support continued exploration of their unique adaptive traits for applications in biotechnology and vaccine storage.

## Data Availability

The datasets presented in this study can be found in online repositories. The names of the repository/repositories and accession number(s) can be found in the article/Supplementary material.
