# Supplementary material for: Antarctic microbial protein-rich extracts with cryoprotective potential for cell and viral preservation
Source: Front Microbiol. 2026 Apr 28;17:1800179. doi: 10.3389/fmicb.2026.1800179 (PMC13163687; doi:10.3389/fmicb.2026.1800179)
Supplement: Supplementary file 1 [file Table_1.docx]

Supplementary Material

**Supplementary Material 1.** Stabilizers used in the study for the cryopreservation of HEK293 cells.

| **Stabilizer** | **Concentration** |
| --- | --- |
| DMSO | 10% |
| AFP III | 1 mg.mL ^-1^ |
| *Pedobacter sp.* BGS4005 | 1 mg.mL ^-1^ |
| *Psychrobacter sp.* P61 | 1 mg.mL ^-1^ |
| *Salinibacterium sp.* P45 | 1 mg.mL ^-1^ |
| Fetal Bovine Serum (negative control) | 40% |

**Supplementary Material 2.** Stabilizers used in Adenovirus viral suspensions.

| **Stabilizer** | **Concentration** |
| --- | --- |
| Glycerol | 2.5% |
| AFP III | 1 mg.mL ^-1^ |
| *Rhodotorula sp.* C01 | 1 mg.mL ^-1^ |
| *Pedobacter sp.* BGS4005 | 1 mg.mL ^-1^ |
| *Psychrobacter sp.* P61 | 1 mg.mL ^-1^ |
| *Salinibacterium sp.* P45 | 1 mg.mL ^-1^ |
| Negative Control | 0.01 M Tris HCl |
