# Supplementary material for: Antarctic microbial protein-rich extracts with cryoprotective potential for cell and viral preservation
Source: Front Microbiol. 2026 Apr 28;17:1800179. doi: 10.3389/fmicb.2026.1800179 (PMC13163687; doi:10.3389/fmicb.2026.1800179)

Supplementary Material - Images

Supplementary Material. Electron microscopy images of the Antarctic isolates used in this study.

Rhodotorula sp. C01

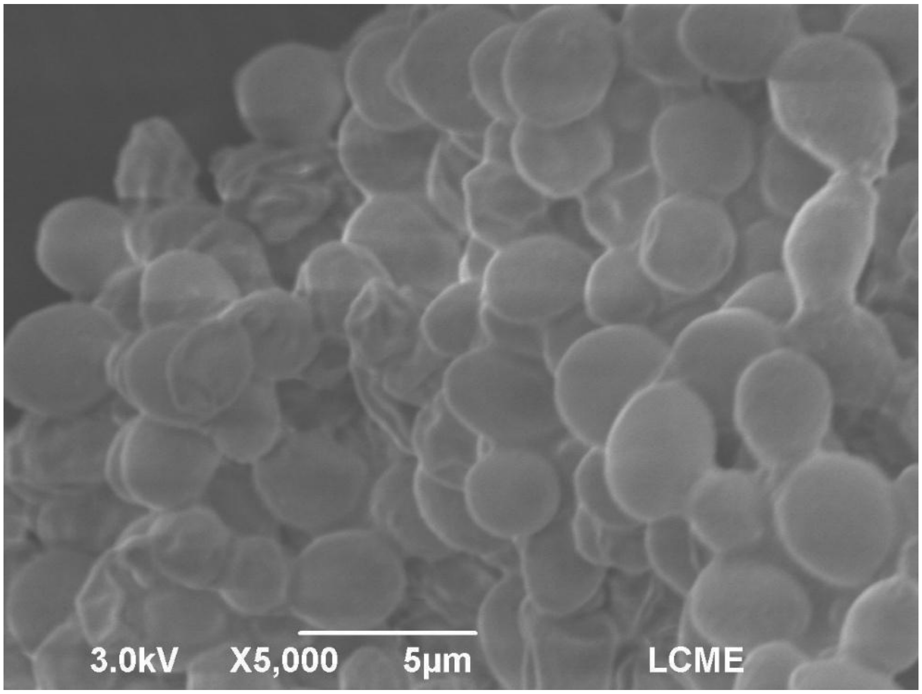

Supplementary Material

Rhodotorula sp. C1001

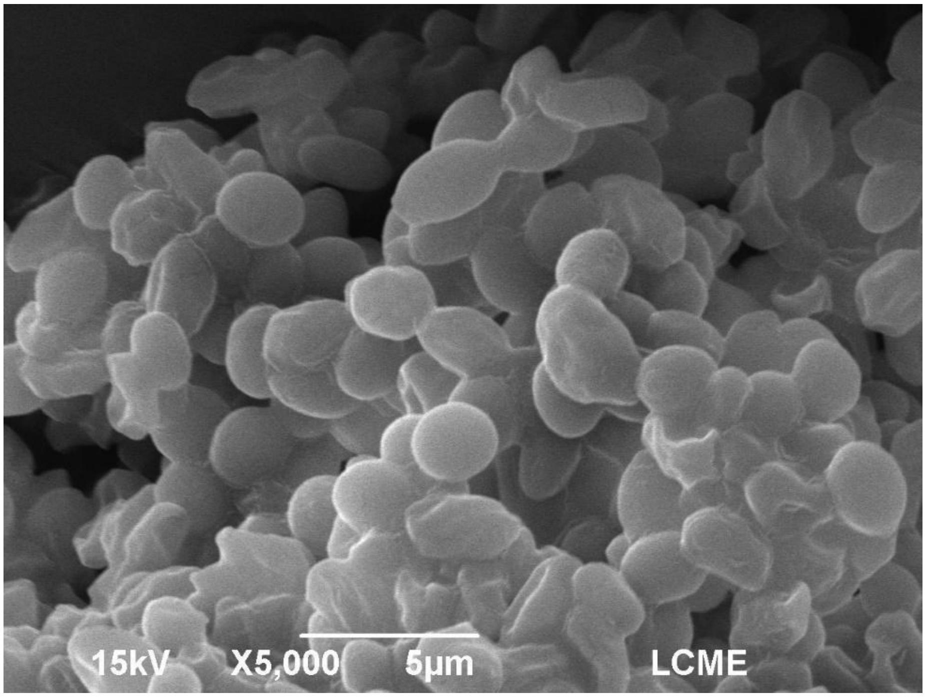

Supplementary Material

Planococcus sp. P25

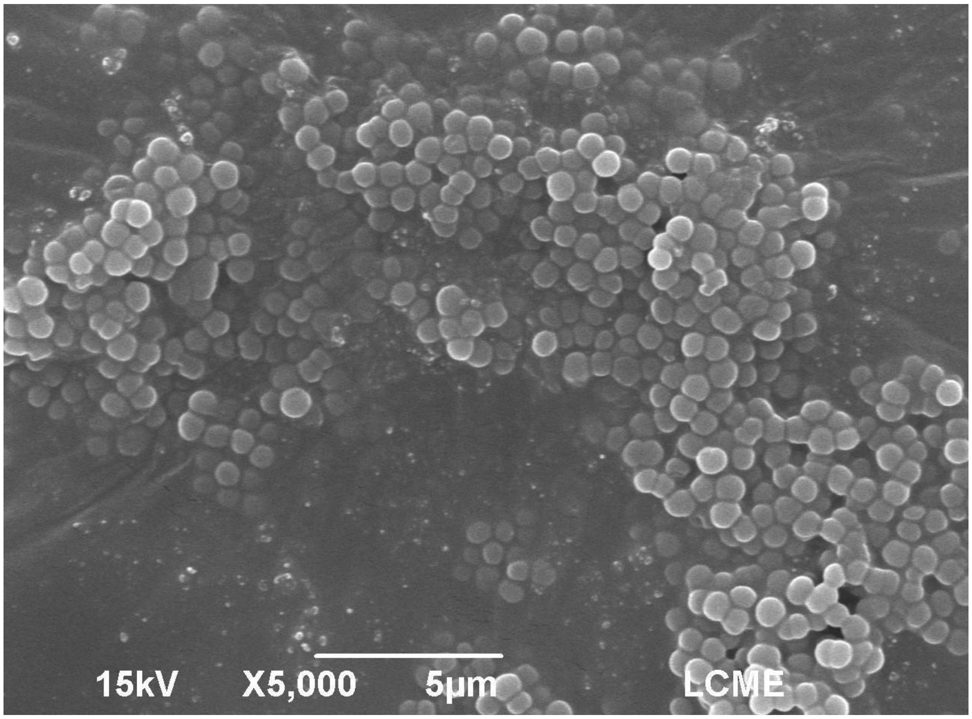

Planococcus sp. P11

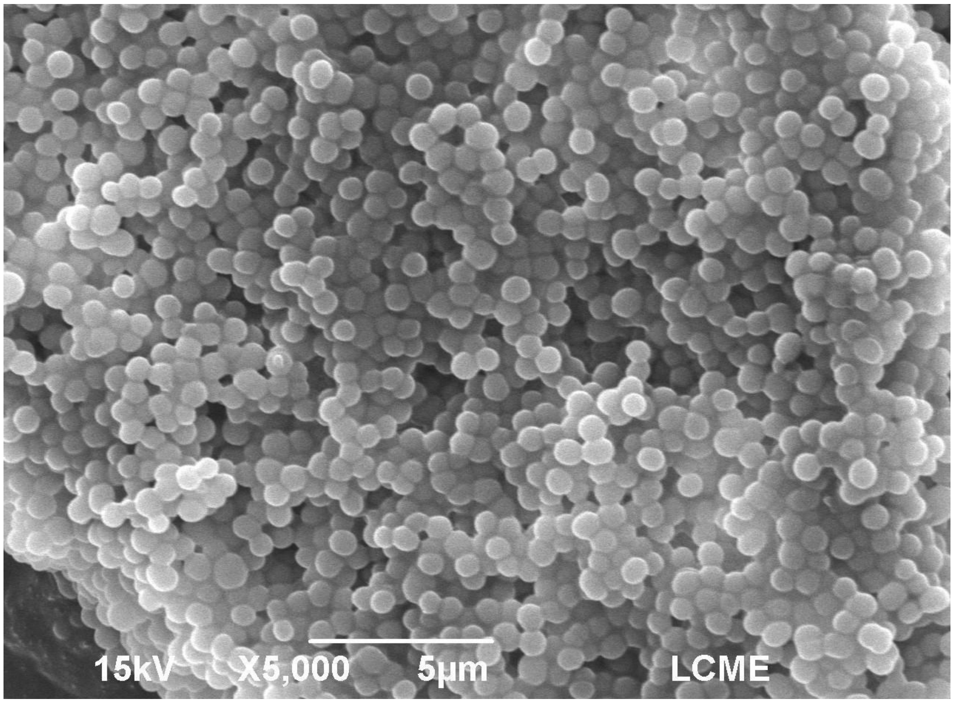

Supplementary Material

Planococcus sp. P18

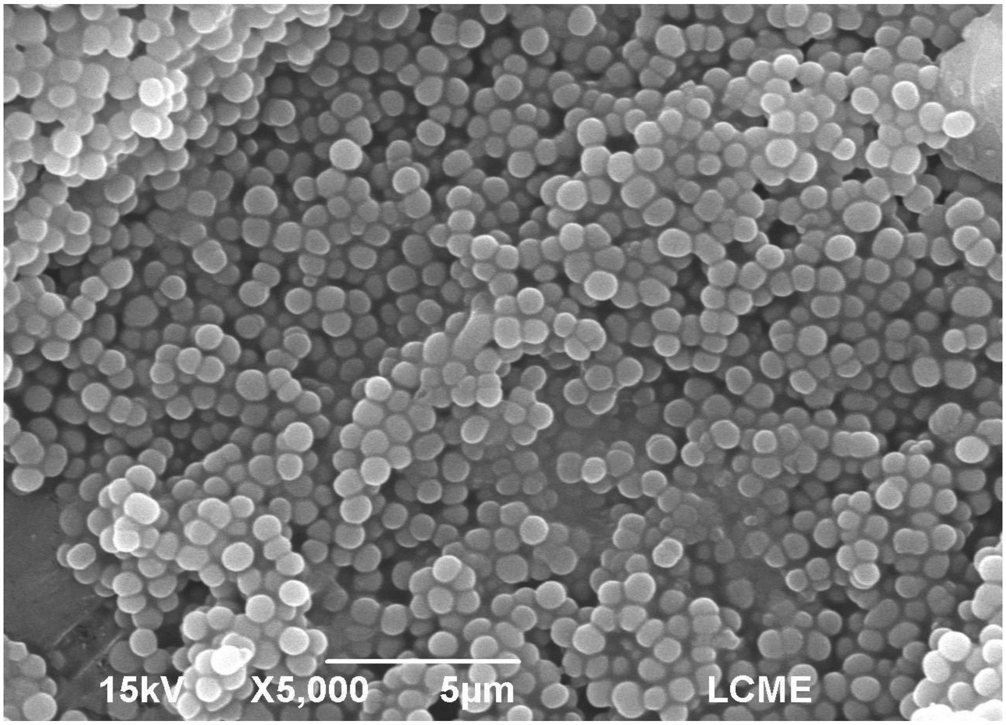

Supplementary Material

Planococcus sp. P7

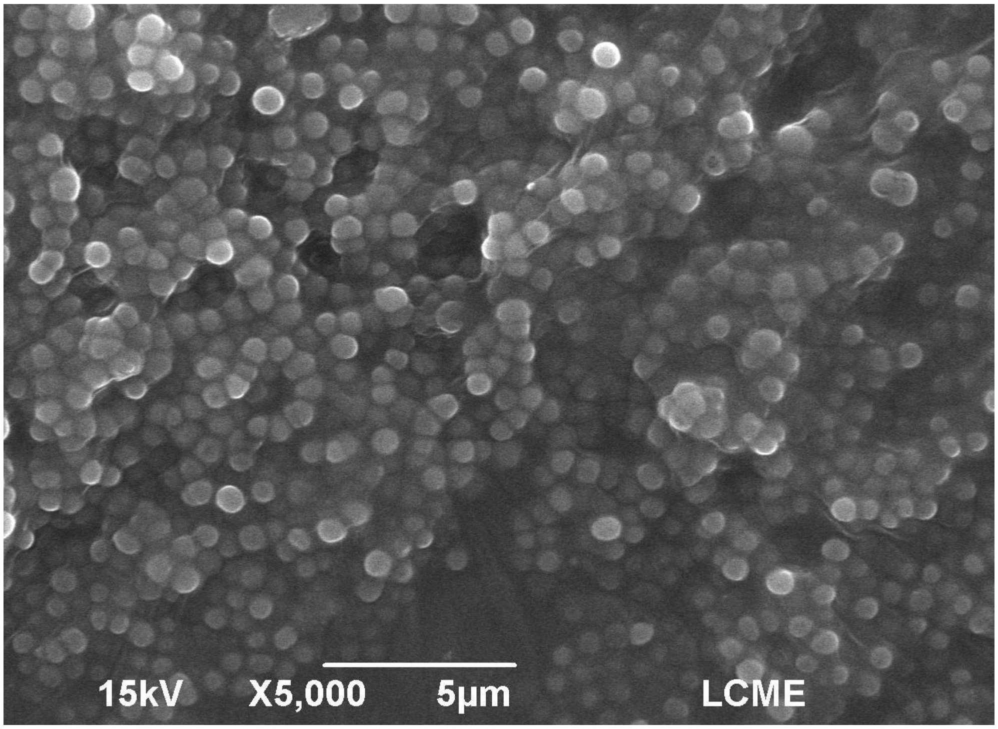

Planococcus sp. P6

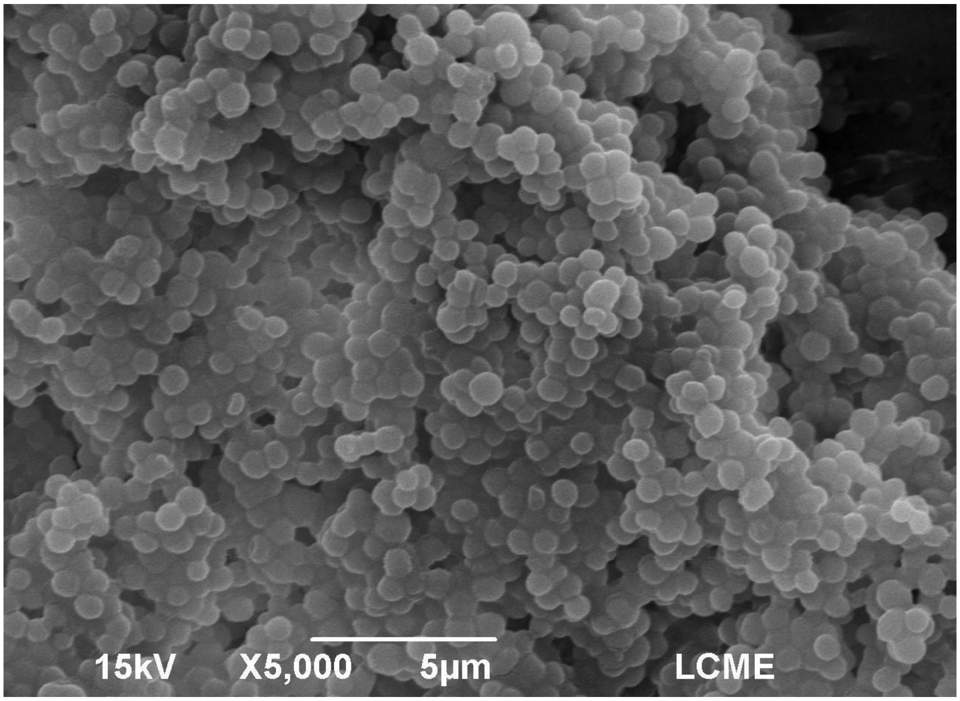

Planococcus sp. P9

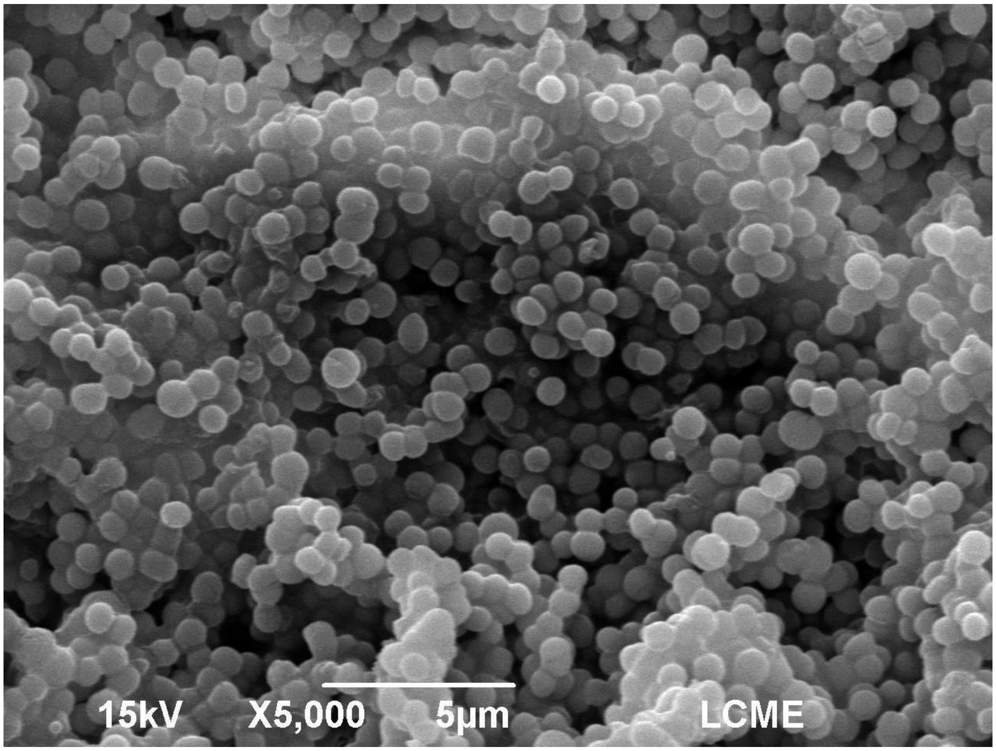

Supplementary Material

Psychrobacter sp. P53

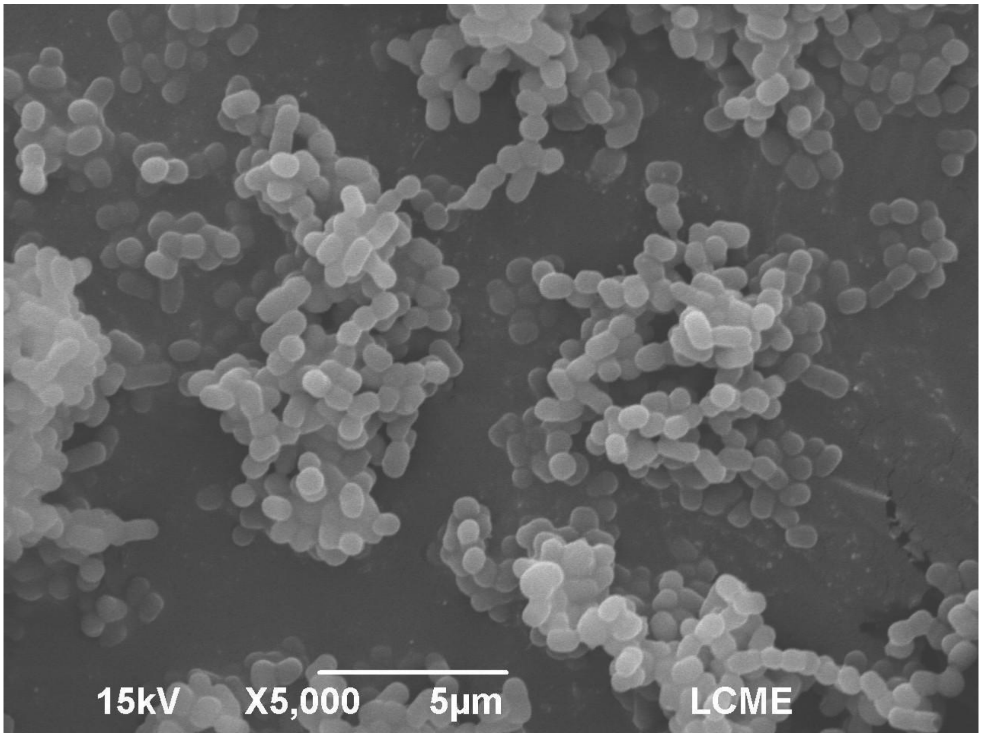

Psychrobacter sp. P61

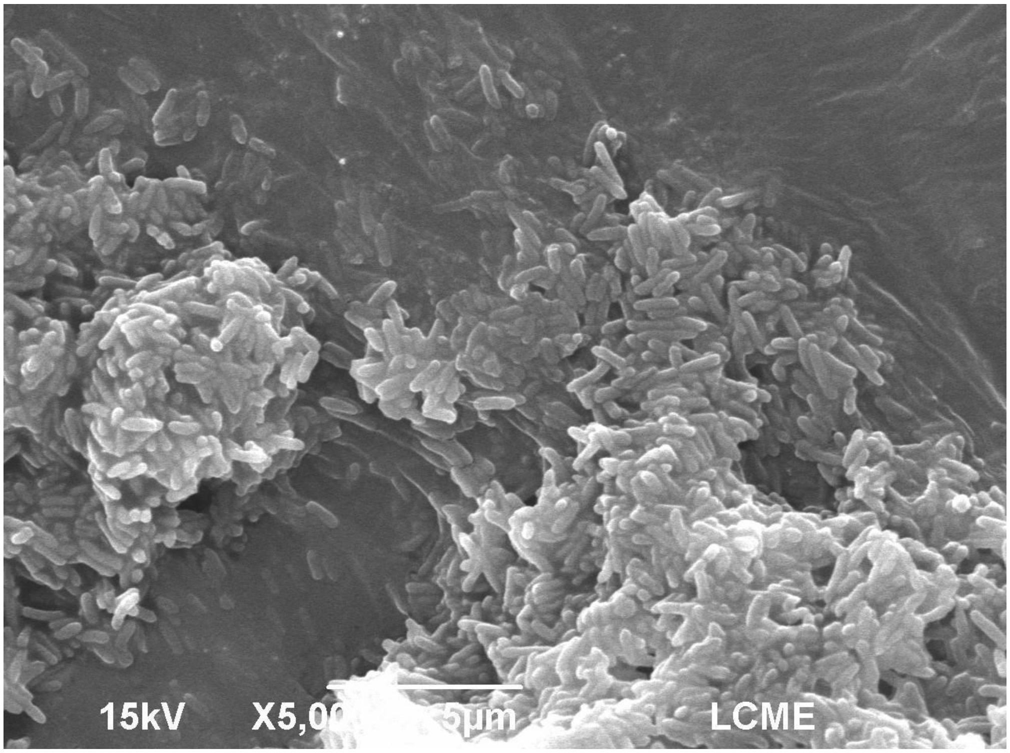

Supplementary Material

Psychrobacter sp. PSC253

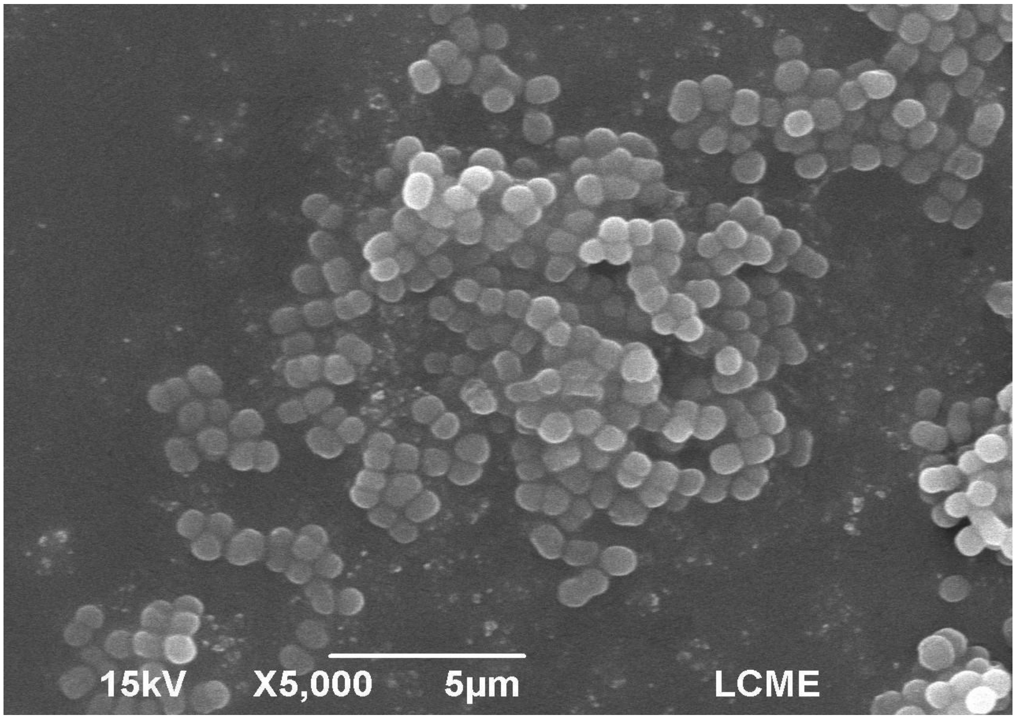

Supplementary Material

Psychrobacter sp. P26

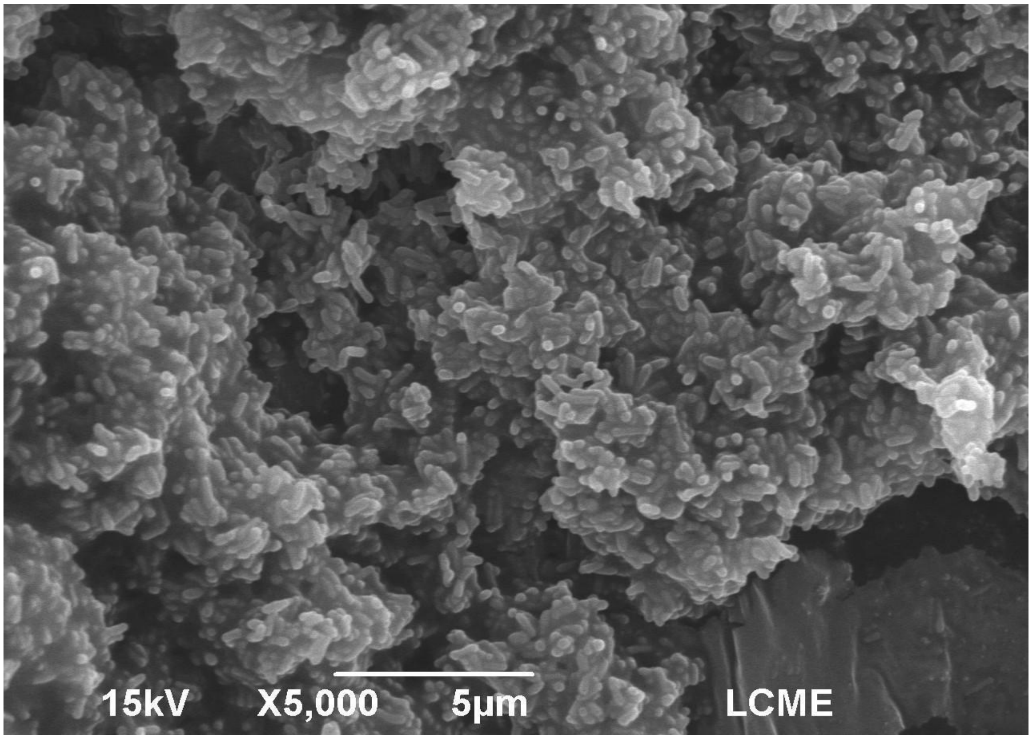

Supplementary Material

Salinibacterium sp. P49

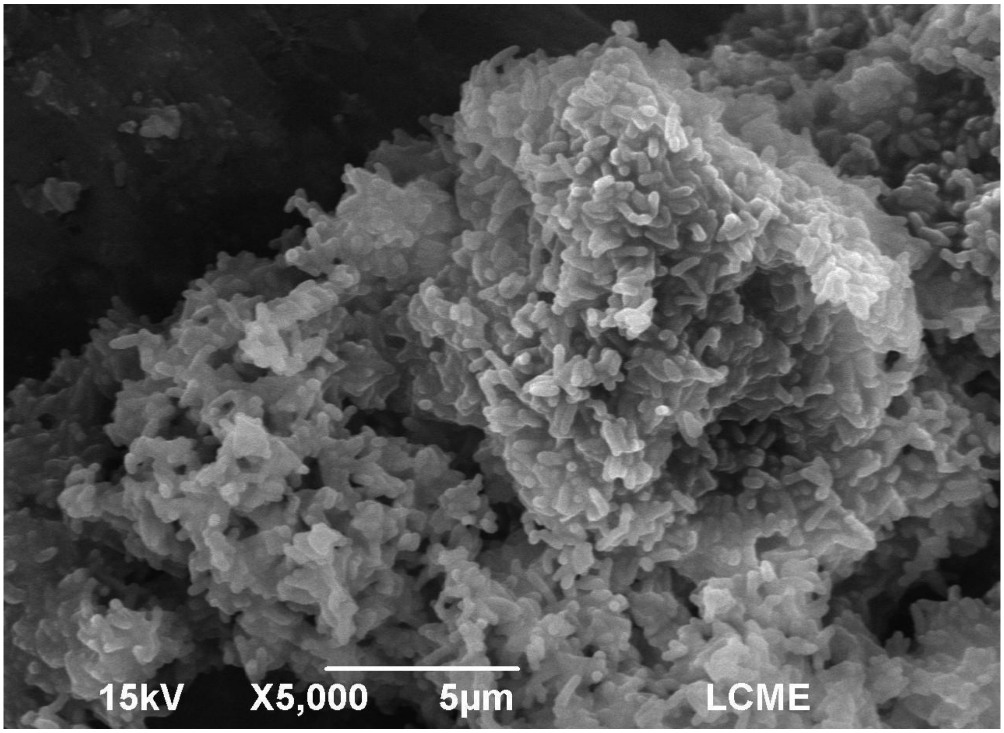

Supplementary Material

Salinibacterium sp. P45

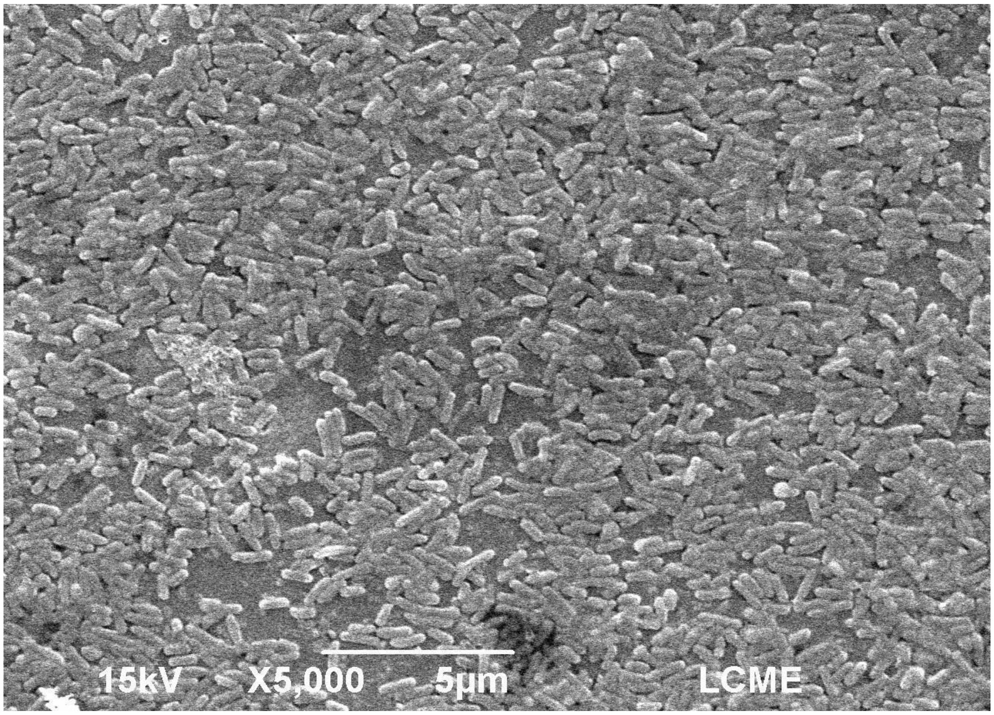

Arthrobacter sp. BGS04

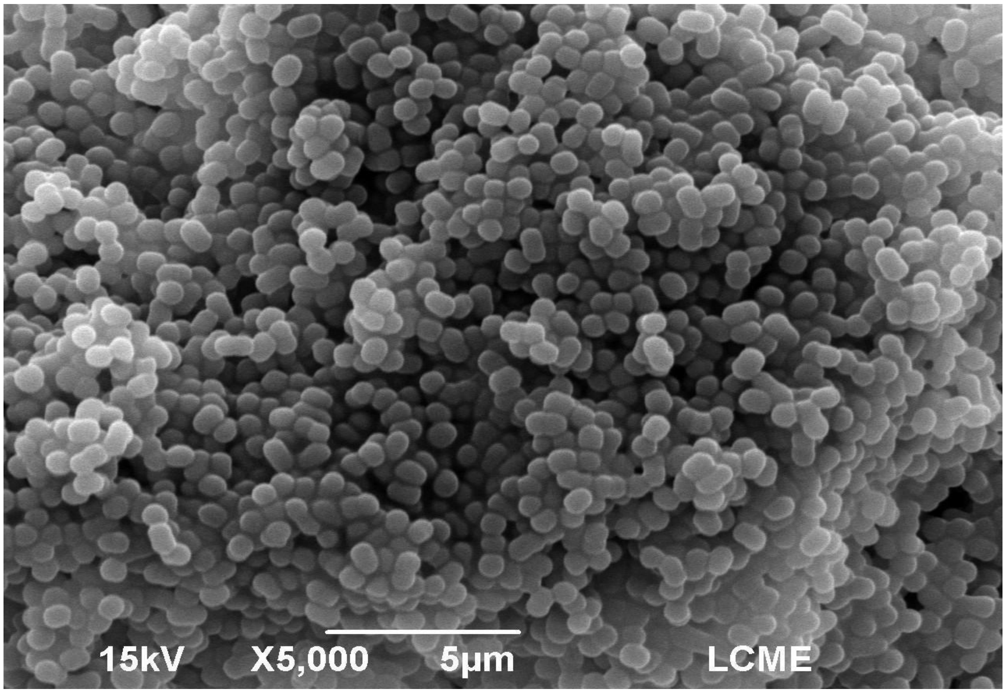

Supplementary Material

Arthrobacter sp. BGS3001

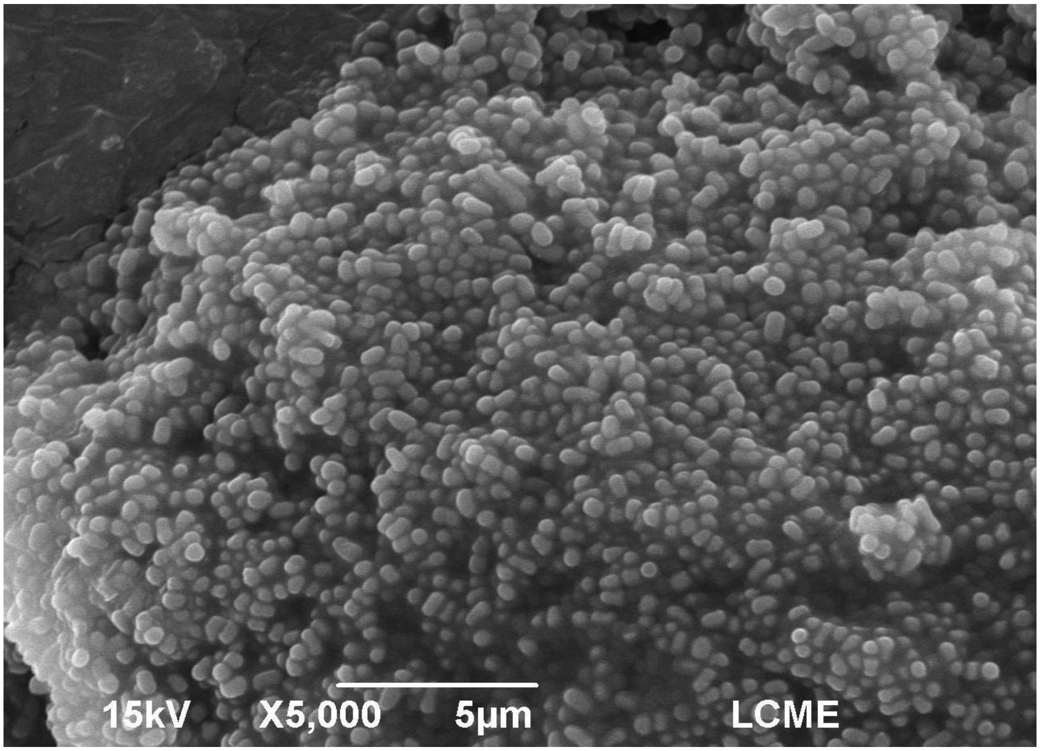

Supplementary Material

Acinetobacter sp. M125C

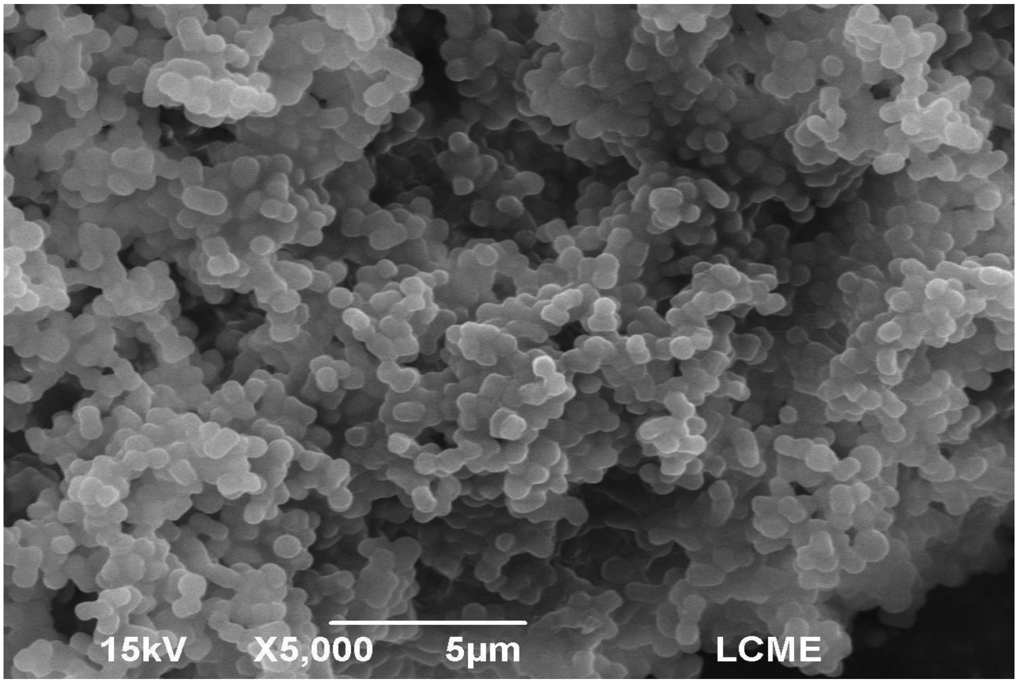

Supplementary Material

Pedobacter sp. BGS4005

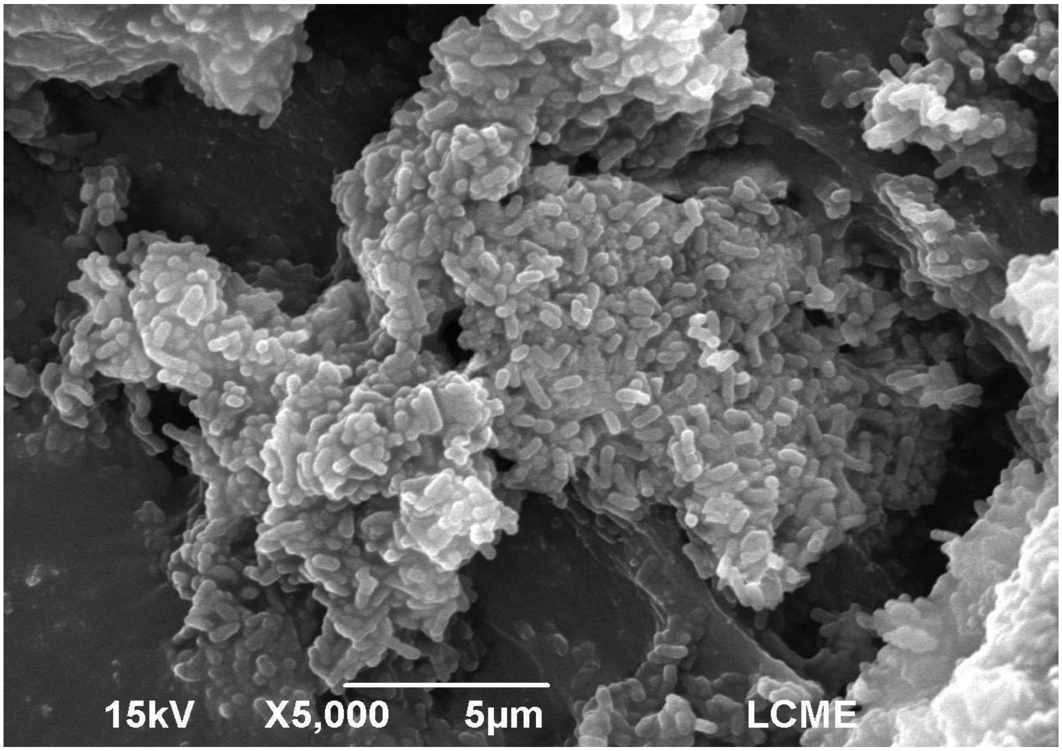

Cryobacterium sp. P64

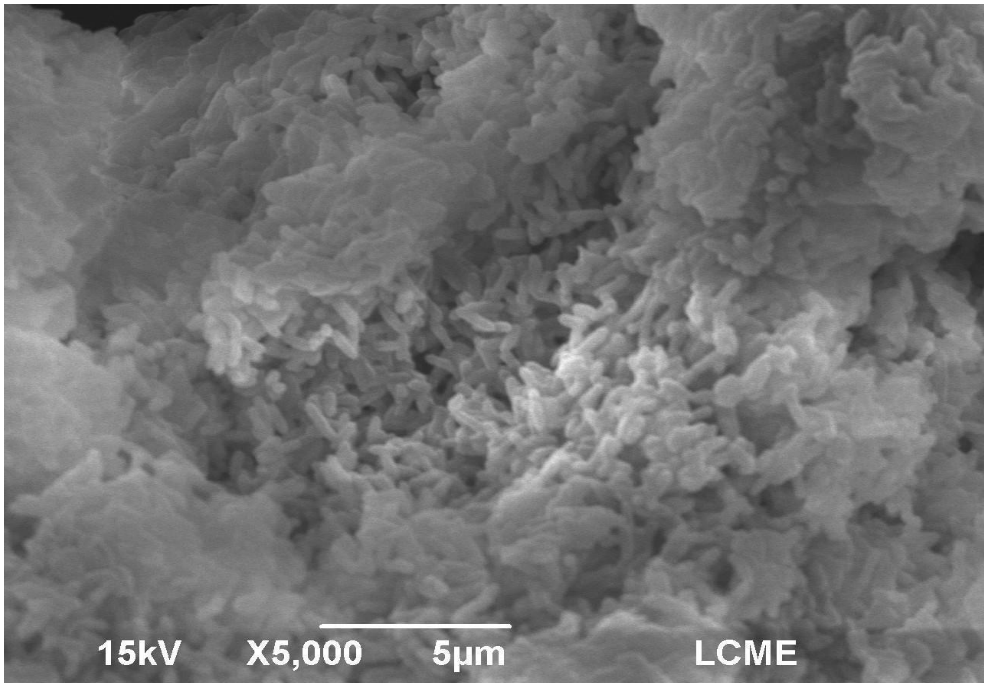

Pseudomonas sp. BGS05

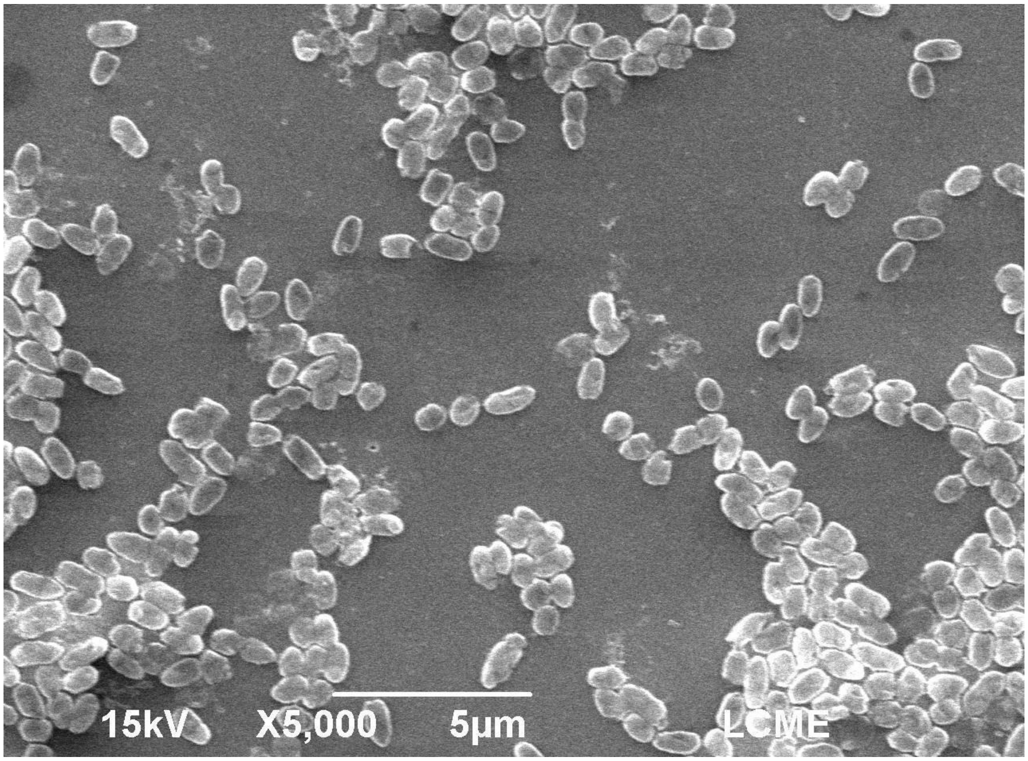

Supplementary Material

Rhodococcus sp. BGS2009L

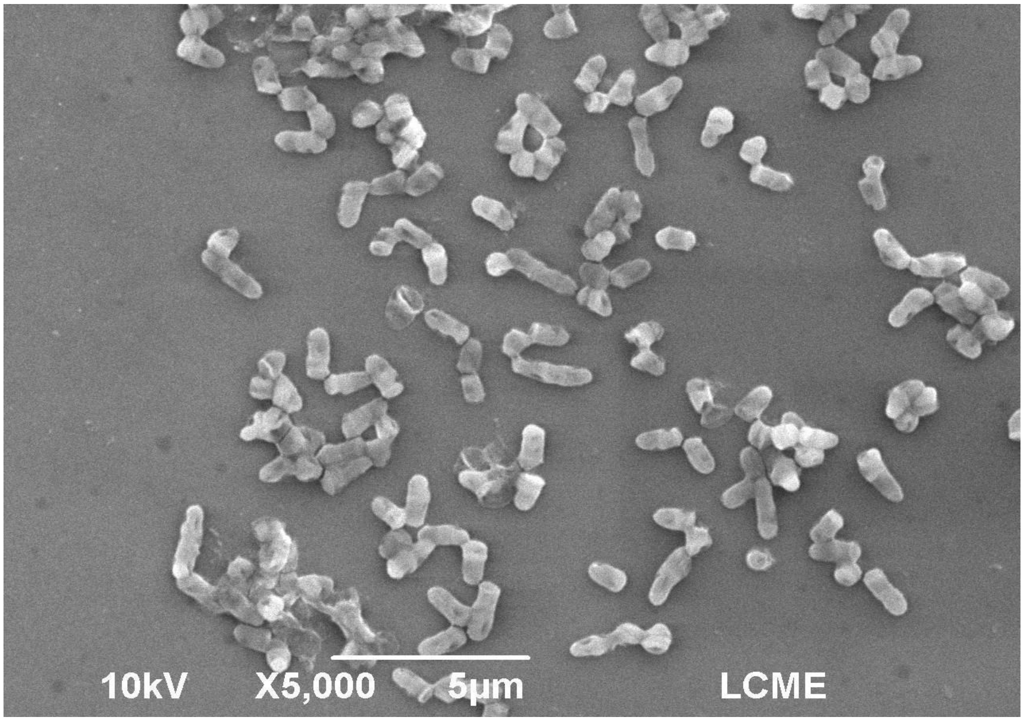

Supplementary Material

Phyllobacterium sp. P31

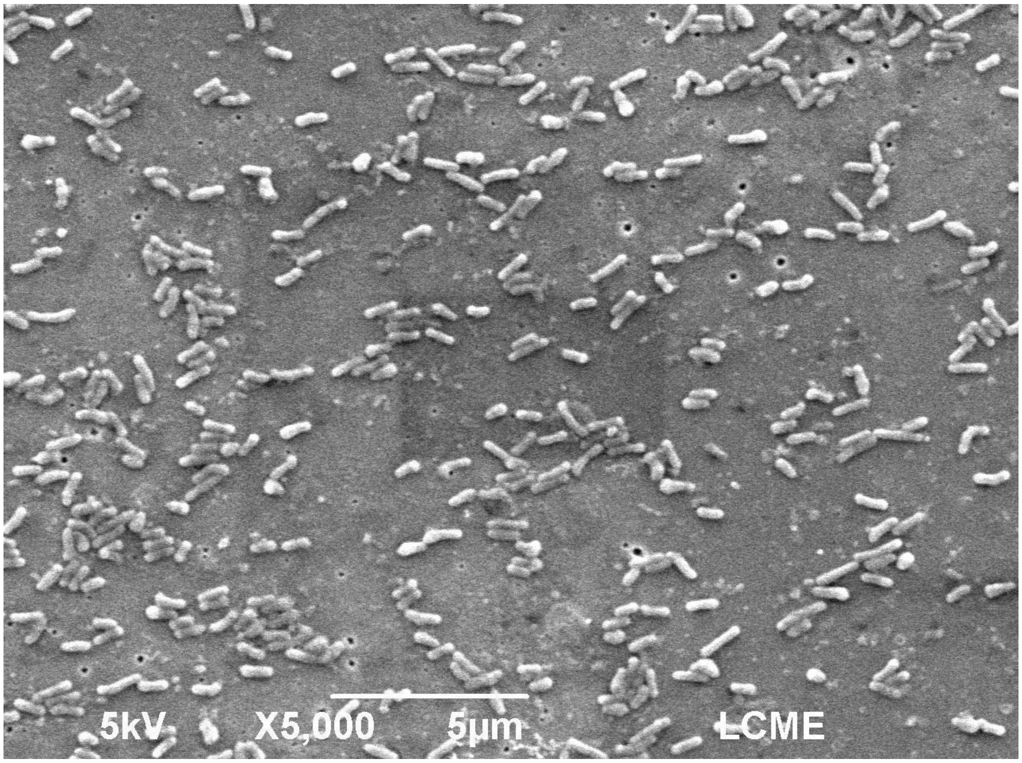

Supplement: Supplementary file 2 [file Supplementary_file_1.pdf]
